# Targeting CB1R Rewires Ca^2+^-Dependent Mitophagy to Promote Nerve Regeneration

**DOI:** 10.7150/thno.119712

**Published:** 2025-08-11

**Authors:** Ningning Wang, Weizhen Li, Tuo Yang, Baolong Li, Chuikai Meng, Xiongyao Zhou, Jialu Sun, Kaiming Yu, Shusen Cui, Rangjuan Cao

**Affiliations:** 1 Department of Hand and Foot Surgery, China-Japan Union Hospital of Jilin University, Changchun 130033, China.; 2 Jilin Provincial Key Laboratory of Peripheral Nerve Injury and Regeneration, Changchun 130033, China.

**Keywords:** Ca^2+^, mitophagy, cannabinoid receptor 1, Schwann cell, nerve regeneration.

## Abstract

**Background:** Ion homeostasis is disrupted following nerve injury, and elevated Ca^2+^ levels have been reported to induce Schwann cell (SC) death. Notably, clinical interventions such as electrical stimulation enhance Ca^2+^ influx and facilitate nerve regeneration. These findings highlight the need to clarify the precise role of Ca^2+^ signaling in nerve regeneration.

**Methods:** We assessed extracellular Ca^2+^ concentrations in both human and murine peripheral nerve tissues after injury. Transcriptomic profiling identified CB1R as a key Ca^2+^-related gene and *in vitro* validation was performed with primary cultured SC and nerve explants. A sciatic nerve crush model was established in SC-specific CB1R knockout mice. Mitophagy, cellular metabolic homeostasis, and axonal regeneration were systematically assessed using proteomics, calcium imaging, and *in vivo* analyses. Additionally, the CB1R antagonist JD5037 was administered in both sciatic and optic nerve injury models to evaluate its translational potential.

**Results:** Peripheral nerve injury (PNI) leads to elevated extracellular Ca^2+^ levels at the injury site, where a moderate increase (~1.5-fold) favors SC survival. PNI also induces upregulation of CB1R, genetic ablation of CB1R enhances Ca^2+^ influx, promotes SC survival, and maintains metabolic homeostasis. Mechanistically, CB1R interference upregulates adenine nucleotide translocase 2 (ANT2) expression, promotes mitochondrial permeability transition pore (mPTP) opening and mitochondrial membrane depolarization, thereby activating PINK1/Parkin-mediated mitophagy. This process improves mitochondrial quality and enhances energy metabolic efficiency, ultimately promoting axonal regeneration and functional recovery. Furthermore, systemic administration of the CB1R antagonist JD5037 similarly enhances regeneration of both peripheral and optic nerves *in vivo*.

**Conclusion:** Moderate extracellular Ca^2+^ elevation establishes a pro-regenerative microenvironment after nerve injury. Targeting CB1R facilitates Ca^2+^ influx, enhances mitophagy via the PINK1/Parkin pathway, and promotes nerve regeneration. These findings identify CB1R as a viable therapeutic target and support the translational potential of JD5037 for nerve injury treatment.

## Introduction

Nerve injuries are associated with high incidence and disability rates worldwide, posing a serious threat to human health and life quality 1-4. Peripheral nerve injuries not only result in sensory and motor dysfunction, but also lead to neuropathic pain, muscle atrophy, and permanent disability if left untreated 1,2. Despite the inherent regenerative capacity of the peripheral nervous system, the clinical outcomes remain suboptimal, and approximately 50% of patients fail to regain complete functional recovery 5, highlighting the urgent need for novel therapeutic strategies. Nerve regeneration refers to the process by which injured neural tissues restore their structure and function through mechanisms such as cellular repair, axonal regrowth, and functional remodeling 6. Although this process is critical for neural recovery, it remains challenged by limited efficacy and mechanistic complexity 3,4. Peripheral nerve injury is common in clinic practice and is widely employed to study the regeneration process 7,8. Investigating the mechanisms of peripheral nerve regeneration not only provides critical insights into the cellular and molecular basis of neural repair but also offers potential therapeutic strategies for promoting regeneration in the central nervous system.

Following nerve injury, ion homeostasis is disrupted and, Schwann cells (SCs) undergo adaptive reprogramming and transform into a repair phenotype, characterized by their capacity to clear necrotic myelin debris, promote axonal regeneration, and remyelinate regenerating axons 8-10. As a pivotal second messenger, calcium ions (Ca^2+^) are extensively involved in cellular stress responses and repair mechanisms 11. Previous studies have consistently suggested that elevated extracellular calcium concentrations induce Schwann cell death, thereby impairing peripheral nerve regeneration 12,13. In contrast, electrical stimulation—widely used in clinical settings—has been shown to promote nerve repair by enhancing intracellular calcium signaling in Schwann cells 11,14-16. This apparent contradiction highlights the need for finely tuned calcium regulation. However, direct manipulation of calcium levels often leads to non-physiological effects, disrupting cellular homeostasis and potentially triggering cytotoxicity or programmed cell death 17. Therefore, targeting upstream signaling molecules, such as calcium channels or calcium-binding proteins, may represent a more physiologically and controllable approach for calcium signaling modulation.

In this study, we unexpectedly observed a moderate elevation in calcium levels in peripheral nerves following injury in both humans and mice, which intriguingly promoted SC function. Focusing on calcium, we identified *Cnr1* (Cannabinoid receptor 1) as a key gene associated with calcium signaling in the injury microenvironment. *Cnr1* encodes cannabinoid receptor type 1 (CB1R), a G-protein coupled receptor known for its high specificity in regulating intracellular calcium homeostasis across various cell types 18-21. Our previous research demonstrated CB1R expression in SCs within the peripheral nervous system, where it mediates abnormal ketone metabolism, leading to metabolic dysfunction and diabetic peripheral neuropathy 22. Building on this foundation, we now show that CB1R acts as a negative regulator of calcium-mediated mitophagy and SC metabolic remodeling during peripheral nerve injury repair. Mechanistically, conditional knockout of CB1R in SCs enhanced Ca^2+^ influx, increased ANT2 expression, and activated the PINK1/Parkin signaling pathway, significantly promoting mitophagy. This enhanced mitophagy facilitated the efficient clearance of damaged mitochondria, improved SC metabolic homeostasis, and markedly accelerated axonal regeneration. In addition, this study evaluated the therapeutic potential of the peripheral CB1R antagonist JD5037 in promoting regeneration of both peripheral and optic nerves.

Collectively, our findings demonstrate that a moderate elevation of Ca^2+^ levels (~1.5-fold) facilitates the formation of a pro-regenerative microenvironment. We propose that targeting the CB1R-Ca^2+^-PINK1/Parkin axis offers a novel metabolism-based strategy to promote nerve repair. Furthermore, our findings highlight JD5037 as a promising therapeutic candidate, providing new pharmacological targets and a solid theoretical foundation for the clinical translation of treatments for nerve injuries.

## Materials and Methods

### Patient samples

Human peripheral nerve samples were obtained from the Department of Hand and Foot Surgery, China-Japan Union Hospital of Jilin University. The use of human tissues in this study was approved by the Ethics Committee of China-Japan Union Hospital of Jilin University (2025071603). Patients with uncontrolled hypertension or metabolic disorders were excluded, as these chronic conditions may independently contribute to neurodegenerative changes. Specifically, peripheral nerve samples were obtained from discarded nerve tissues excised during surgery in patients with severe peripheral nerve injury (within 6 h post-injury, including ulnar and digital nerves), as well as from digital nerves collected during digit amputation in patients with congenital polydactyly. Residual nerve segments (~2 cm) from the amputated limbs were rapidly snap-frozen in dry ice to minimize injury-induced artifacts. These samples were subsequently processed for calcium quantification using a colorimetric calcium assay kit. Parallel tissue aliquots were used for protein extraction and subsequent Western blot according to the established experimental protocols. A summary of patient characteristics included in this study is provided in Table S1.

### Animals

All animal experiments were conducted in accordance with protocols approved by the Animal Ethics and Welfare Committee of Jilin University, in accordance with the National Institutes of Health Guide for the Care and Use of Laboratory Animals (20230609-01). Mice were housed under specific-pathogen-free (SPF) conditions, with no more than five animals per cage, maintained on a 12-h light/dark cycle at a constant ambient temperature of 23 °C. Animals had *ad libitum* access to standard laboratory chow (Diet 7097, Harlan Teklad) and water. All genetically modified mouse strains used in this study were on a C57BL/6 background. *Cnr1^fl/fl^* mice were kindly provided by Prof. Feng Wang (University of Chinese Academy of Sciences), and *PLP-Cre^ERT2^* mice were obtained from OBiO Technology (strain ID: C001031). Wild-type male C57BL/6 mice (8 weeks old, 18-22 g) were purchased from Changsheng Biotechnology (China).

### Key Resources

Key resources including antibodies, sequences, chemicals, peptides, recombinant proteins, and reagents were listed in Tables S2-S5 (Supporting Information).

### Genotyping

Genotyping was performed using PCR amplification of genomic DNA extracted from tail or toe tissue collected from neonatal mice (within 7 days after birth). Tissue samples were incubated in 75 μL lysis buffer containing 25 mM NaOH and 0.2 mM EDTA at 98 °C for 1 h with constant shaking. The lysate was then cooled to 15 °C, followed by neutralization with an equal volume of 40 mM Tris-HCl buffer (pH 5.5). The resulting DNA extract was used directly as the template for genotyping PCR.

### Sciatic nerve crush

All experiments were performed on adult male C57BL/6 mice (8 weeks old, 18-22 g). Animals were randomly assigned to experimental groups. Mice were anesthetized using isoflurane (5% for induction, 2% for maintenance), and the surgical area (hind limbs and lower back) was shaved and disinfected with iodine. Ophthalmic ointment was applied to prevent corneal drying. A skin incision was made over the mid-thigh, and the biceps femoris and gluteus superficialis muscles were bluntly separated to expose the sciatic nerve. A standardized sciatic nerve crush (SNC) injury was created by applying constant pressure with 5-mm surgical forceps (J31060, Inox Electronic) orthogonally to the nerve for 30 s, approximately 20 mm distal to the dorsal root ganglion (DRG) 23. The incisure was sutured and animals were monitored closely until they woke up completely and were able to eat and drink properly. Pain management was conducted by adding Metamizole (500 mg per 100 mL drinking water) from 1 day before the crush until 3 days post crush.

### Optic nerve crush

Anesthetized adult male mice underwent optic nerve crush. Briefly, a spring scissor was used to incise conjunctiva, and blunt forceps were employed to create a visualized pocket infraorbital. The exposed optic nerve was crushed with fine forceps for 10 s at around 1 mm behind the optic disc, taking care to avoid injury to the ophthalmic artery. During the surgery, ophthalmic ointment was applied to protect the corneal from drying 24.

### Intravitreal injection

After anesthetizing the mice, they were secured in a stereotaxic apparatus. Intravitreal injection was performed using a micromanipulator, with careful attention to avoid damaging the lens to eliminate the possibility of false-positive nerve regeneration. JD5037 (10 μM, 1 μL) was injected into the vitreous chamber. After the injection, the injection site of the eyeball was covered with antibiotic ointment to prevent bacterial infection, and the mice were maintained on a warm platform throughout the entire surgical procedure.

### Tamoxifen administration

For inducible Schwann cell-specific *Cnr1* knockout, *Cnr1^fl/fl^*; *PLP-Cre^ERT2^* mice and littermate controls (*Cnr1^fl/fl^*) received intraperitoneal injections of tamoxifen (100 mg/kg; dissolved in corn oil and ethanol, 9:1) every other day for a total of three injections 25.

### JD5037 administration

JD5037 was dissolved in a vehicle solution containing 10% DMSO, 40% PEG300, 5% Tween-80, and 45% saline. JD5037 at the concentration of 3 mg/kg was administrated via oral gavage for 14 consecutive days post-injury. Control groups received equal volumes of either vehicle or sterile saline.

### ω-conotoxin treatment

Primary Schwann cells were treated with ω-Conotoxin GVIA/MVIIC (Aladdin, Cat#274837) at a final concentration of 1 μM for 30 min after siRNA interference. The toxin mixture inhibits N-, P-, and Q-type calcium channels. Control groups received an equal volume of vehicle (DMSO).

### Behavioral tests

#### DigiGait walking analysis

Gait performance was evaluated using the automated DigiGait system (Mouse Specifics) under standardized conditions. Mice were placed on a transparent motorized treadmill set at a constant speed of 20 cm/s. A high-speed video camera located beneath the treadmill recorded the paw movements during locomotion. Prior to baseline assessment, animals were trained for three consecutive days to ensure acclimatization and the ability to maintain consistent walking for at least 6 s. Following a 1-day rest, surgery was performed, and gait analysis was conducted at the indicated postoperative time points. During testing, recordings were acquired once each mouse maintained uninterrupted walking at 20 cm/s for a minimum of 5 s. Sciatic Functional Index (SFI) was calculated using DigiGait software 26.

#### Footprint analysis

To evaluate gait recovery, footprint analysis was performed using a custom-made runway measuring 80 cm in length and 10 cm in width, with a dark chamber containing food placed at the end. Prior to testing, mice were trained to traverse the corridor. The plantar surfaces of the hind paws were coated with non-toxic ink, and animals were allowed to walk freely across a white paper-lined floor. At least three uninterrupted, clear sets of footprints were collected per mouse. The SFI was calculated based on footprint parameters 27.

(EPL: experimental print length; NPL: normal print length; ETS: experimental toe spread; NTS: normal toe spread; NNL: normal narrow length; NTL: normal toe length.)



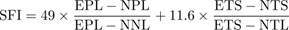



#### Ladder rung walking test

Fine motor coordination was assessed using a horizontal ladder rung walking apparatus. The setup consisted of 1-m-long, 20-cm-high Plexiglas sidewalls and removable rungs (25 mm diameter) with a minimum inter-rung spacing of 1 cm. The ladder was elevated 30 cm above the ground with a goal box at the end. Mice were trained to cross the apparatus with regularly spaced rungs, followed by testing on an irregular rung pattern to assess adaptive locomotion. Two high-resolution cameras were positioned diagonally at both halves of the ladder to record foot placement. Scoring was performed based on paw placement accuracy as follows 28,29: [Full Placement: complete paw placement on rung without error (score = 6); Partial Placement: partial contact, stable (score = 4); Slip: paw slips off the rung (score = 2); Miss: paw completely misses the rung (score = 0)]. Performance was quantified by total score per step and percentage of missed steps:



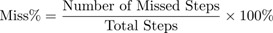



#### Twitch force measurement

Twitch force of the hindlimb muscles was measured using a dual-mode muscle lever system (Aurora Scientific, model 1300A). Mice were anesthetized and placed on a 37 °C heating pad. The right foot was secured onto a footplate connected to the force transducer, while the left knee was gently stabilized using knee clips to minimize movement. The footplate angle was set to a default of 17°, with slight adjustments made to optimize force generation. Muscle contractions were induced via direct electrical stimulation using two needle electrodes inserted into the skin near the tibialis anterior muscle. A constant current of 2 mA and a pulse width of 0.2 ms were applied throughout the experiment. Twitch force values were normalized to the body weight of each animal 30.

### Muscle wet weight assessment

Under isoflurane anesthesia, the tibialis anterior (TA) and gastrocnemius (GA) muscles on the injured side were carefully dissected and immediately weighed using an electronic balance (Mettler-Toledo, USA). After weighing, the mice were further deeply anesthetized and perfused for subsequent histological analysis 8.

### Immunofluorescence staining

For sciatic nerve analysis, mice were deeply anesthetized with isoflurane, and nerves were harvested and post-fixed in 4% paraformaldehyde (PFA) at 4 °C for 1 h, followed by cryoprotection in 30% sucrose for ≥ 3 days. Tissues were embedded in Tissue-Tek OCT compound, stored at -80 °C, and sectioned at 10 μm thickness. Sections were rinsed with PBS, permeabilized and blocked in 5% donkey or goat serum with 0.5% Triton X-100 for 2 h at room temperature. Coronal sections were stained with CB1R (1:500, rabbit, Abcam) and S100β (1:300, mouse, Sigma), while sagittal sections were stained with Tuj1 (1:1000, mouse, Abcam), a marker of regenerating axons. The nerve crush site was identified by axonal disruption and peak Tuj1 intensity. After washing with PBST (0.5% Tween-20), sections were incubated with fluorophore-conjugated secondary antibodies for 2 h at room temperature. Tuj1 fluorescence intensity was quantified at 500 μm intervals distal to the crush site and normalized to the proximal segment. Four to six sections per mouse were analyzed using a confocal microscope (Nikon A1R) at 10×magnification.

For neuromuscular junction (NMJ) staining, the extensor digitorum longus (EDL) muscle was isolated and fixed in 4% PFA at 4 °C overnight, then washed in 0.1 M glycine/PBS for 30 min. Samples were blocked in buffer containing 2% Triton X-100, 5% BSA, and 5% goat serum for 2 h at room temperature. Primary antibodies against Synapsin-1 (1:300, rabbit, CST), neurofilament light chain (NFL, 1:500, rabbit, CST), and α-bungarotoxin (α-BTX, 1:1000) were applied overnight at 4 °C. After PBS washes, fluorophore-conjugated secondary antibodies were applied overnight. Images were captured using a Nikon A1R confocal microscope. Quantification included analysis of fragmented endplates, mono-innervated junctions, and acetylcholine receptor (AChR) aggregation.

For flat-mounted mouse retinas, the eyes were enucleated and the retinas were carefully dissected. After removing the vitreous body, the retinas were fixed in 4% paraformaldehyde for 20-30 minutes, followed by three washes in PBS, each for 10 minutes. The retinas were then cut into a cross shape and flat-mounted onto glass slides with the ganglion cell layer facing upward. To block nonspecific binding and improve tissue permeability, the samples were incubated in a blocking solution containing 5% normal goat serum and 0.5% Triton X-100 at room temperature for 2 h. Subsequently, the retinas were incubated with the primary antibody Tuj1 (1:1000, mouse, Abcam) diluted in blocking buffer at 4 °C for 48 h in the dark. After incubation, the tissues were washed four times with PBS containing 0.3% Triton X-100, 15 minutes each. Alexa Fluor-conjugated secondary antibody (1:500) was then applied for 2 h at room temperature in the dark, followed by three additional 10-minute washes in PBS. Finally, the retinas were mounted with anti-fade mounting medium, covered with coverslips, stored in the dark, and imaged using a Nikon A1R confocal microscope to visualize the morphology and distribution of retinal ganglion cells.

### CTB injection and staining

To assess retrograde axonal transport, mice received intramuscular injections of cholera toxin subunit B (CTB, 3 μL, 0.25 mg/mL) into the tibialis anterior of the injured side. Seven days post-injection, animals were transcardially perfused and fixed. Lumbar dorsal root ganglia (L3-L5) were carefully dissected and processed for immunofluorescence staining. CTB-labeled neurons were visualized using a confocal laser scanning microscope (Nikon A1R). The number of CTB-positive neurons was quantified using ImageJ software.

### Transmission Electron Microscopy (TEM)

For ultrastructural analysis, mice were euthanized and sciatic nerves were excised and fixed in 2.5% glutaraldehyde at 4 °C overnight, followed by postfixation in 1% osmium tetroxide. Samples were rinsed in phosphate buffer, dehydrated through a graded ethanol series, and embedded in epoxy resin. Ultrathin sections were prepared using an ultramicrotome (LEICA EM UC7), mounted onto copper grids, and stained with 1% uranyl acetate and lead citrate. Images were acquired using a JEOL transmission electron microscope. Myelin sheath thickness was quantified using the *g*-ratio (axon diameter/fiber diameter). Both myelinated and unmyelinated axons were counted within standardized fields of view.

### *Ex vivo* sciatic nerve explant culture

Sciatic nerves were isolated from adult mice, and the epineurium and perineurium were carefully removed under a stereomicroscope using fine micro-forceps. Nerve explants were cultured in 2 mL of *ex vivo* medium consisting of DMEM/F12 supplemented with 17.5 mM glucose, 0.5 mM pyruvate, 1× GlutaMAX, 1× penicillin/streptomycin, and 1× N2 supplement. Cultures were maintained at 37 °C in a humidified incubator with 5% CO₂. The experimental groups were treated with BAPTA to deplete calcium ions, followed by different concentrations of CaCl_2_ to establish a Ca^2+^ concentration gradient. Media were refreshed every 3 days. After 6 days, explants were collected for immunofluorescence staining.

### Cell culture

Primary Schwann cells were isolated from the sciatic and brachial plexus nerves of 2-day-old rats. Nerves were minced and digested with 3 mg/mL collagenase (Sigma, C0130) at 37 °C for 30 min, followed by 0.25% trypsin (Sigma, T4049) digestion for 8 min at 37 °C. Dissociated cells were cultured in DMEM/F12 medium supplemented with 10% fetal bovine serum (FBS), 100 IU/mL penicillin, and 100 μg/mL streptomycin in a humidified incubator (37 °C, 5% CO_2_). To eliminate fibroblasts, cells were treated with 10 μM cytosine β-D-arabinofuranoside (Sigma, C1768), polyclonal anti-Thy1.1 antiserum (1:1000, Sigma, M7898), and rabbit complement (Millipore, 234400) for immunoselective depletion.

### Cell immunofluorescence staining

Schwann cells were seeded on glass coverslips and fixed with 4% paraformaldehyde for 15 minutes at room temperature. Cells were then permeabilized with 0.2% Triton X-100 in PBS for 10 minutes and blocked with 5% BSA for 1 h to minimize nonspecific binding. Subsequently, cells were incubated overnight at 4 °C with primary antibodies against LC3B (1:200, mouse, CST) and TOM20 (1:200, rabbit, Proteintech) diluted in blocking solution. After washing with PBS, the cells were incubated with appropriate fluorescent-conjugated secondary antibodies (1:500) for 1 h at room temperature in the dark. Nuclei were counterstained with DAPI (1 μg/mL) for 5 minutes. Coverslips were mounted using antifade mounting medium and imaged using a confocal laser scanning microscope (Nikon A1R). Colocalization analysis between LC3B and TOM20 was performed using ImageJ software with the JACoP plugin to quantify mitophagic activity.

### Calcium imaging using Rhod-2 AM

Mitochondrial Ca^2+^ levels were measured using Rhod-2 AM (Abcam, ab142780). Cells were incubated with 5 μM Rhod-2 AM in glass-bottom culture dishes at 37 °C for 30 min in the dark. Following incubation, cells were rinsed with DMEM and imaged using a Nikon A1R laser-scanning confocal microscope (excitation/emission: 549/578 nm). Time-lapse recordings were acquired every 1 min for 8 min of observation under identical imaging settings. Fluorescence intensity was quantified using ImageJ software.

### Calcium Imaging Using Fluo-4 AM

Intracellular free calcium ([Ca^2+^]_i_) levels were evaluated using Fluo-4 AM (Elab Science, E-BC-F100). Schwann cells were loaded with 5 μM Fluo-4 AM diluted in serum-free DMEM/F12 and incubated for 30 min at 37 °C in the dark. Cells were then washed three times with calcium-free Hank's Balanced Salt Solution (HBSS) and equilibrated for 10 min prior to imaging. Fluorescence was captured using a Nikon A1R confocal microscope (excitation/emission: 488/520 nm). Time-lapse recordings were acquired every 1 min for 8 min of observation under identical imaging settings. Quantitative analysis was performed using ImageJ.

### siRNA transfection

Small interfering RNAs targeting *Cnr1* and *Ant2*, as well as a nonspecific negative control siRNA (Integrated Biotech Solutions), were transfected into Schwann cells using Lipofectamine 3000 (Invitrogen, L3000150) according to the manufacturer's instructions. After 6 h of transfection, the medium was replaced with fresh DMEM/F12 containing 10% FBS, and cells were incubated for an additional 72 h prior to downstream analysis. The negative control siRNA consisted of a scrambled sequence with no known target.

### Mitochondrial membrane potential assessment

Mitochondrial membrane potential (ΔΨm) was evaluated using JC-1 dye (Beyotime, C2006). Schwann cells were seeded in glass-bottom confocal dishes and subjected to the indicated treatments. Cells were incubated with 1 μM JC-1 at 37 °C for 30 min in the dark. Following three washes with pre-warmed PBS, fluorescent images were acquired immediately using a Nikon A1R confocal microscope. The ratio of red (J-aggregates) to green (monomers) fluorescence was used as an indicator of ΔΨm.

### Intracellular ROS evaluation

Intracellular reactive oxygen species (ROS) levels were assessed using the DCFH-DA probe (Beyotime, S0033s). Cells cultured in confocal dishes or 6-well plates were incubated with 3 μM DCFH-DA at 37 °C for 30 min in the dark. After washing, images were obtained using a confocal microscope. Quantification of fluorescence intensity was performed using ImageJ software.

### Cell viability assay

Cell viability was assessed using the Cell Counting Kit-8 (CCK-8; DoJinDo, CK04). Schwann cells were seeded into 96-well plates and treated with varying concentrations of Ca^2+^ for 24 h. Subsequently, 10 μL of CCK-8 reagent was added to each well, and the cells were incubated at 37 °C in the dark for 2 h. Absorbance at 450 nm was measured using a microplate reader. Data were presented as mean ± SD from triplicate wells.

### Western blot analysis

Primary Schwann cells, sciatic nerve tissues or surgically discarded human injured nerve tissues were lysed in RIPA buffer containing a protease inhibitor cocktail (Sangon Biotech, C50008). Protein concentrations were determined using the BCA Protein Assay Kit (Beyotime, P0010). Equal amounts of protein were separated by 10% SDS-PAGE and transferred to PVDF membranes. Membranes were blocked in 5% non-fat milk for 1.5 h at room temperature and incubated overnight at 4 °C with primary antibodies. After washing, membranes were incubated with HRP-conjugated secondary antibodies (1:1000; Beyotime) for 2 h at room temperature. To strip and re-probe membranes, Western Blot Antibody Stripping Solution (Epizyme) was applied for 20 min at room temperature, followed by TBST washing and re-blocking. Immunoreactive bands were visualized using chemiluminescence and scanned with a GS800 densitometer. Band intensities were quantified using ImageJ and normalized to loading controls. Experiments were independently repeated four times, and representative results are shown.

### Calcium quantification

Calcium concentrations in mouse and surgically discarded human injured nerve tissues was quantified using a colorimetric calcium assay kit (Beyotime, S1063S) following the manufacturer' s instructions. Fresh tissue or cell samples were lysed using the provided lysis buffer. After complete lysis, samples were centrifuged at 4 °C for 5 min (10,000-14,000 *g*), and the supernatant was collected. Aliquots of the supernatant were added to a 96-well microplate (five replicates per group), followed by addition of the working reagent. After 5 min of incubation in the dark at room temperature, absorbance was measured at 575 nm using a microplate reader. Calcium concentrations were calculated from a standard curve.

### Mitochondrial Permeability Transition Pore (mPTP) assay

The opening of the mitochondrial permeability transition pore (mPTP) was assessed using a fluorescence-based mPTP assay kit (Beyotime, C2009S) according to the manufacturer' s protocol. Briefly, Schwann cells were washed twice with pre-warmed PBS and incubated with Calcein AM at 37 °C for 45 min in the dark. The staining solution was then replaced with fresh culture medium containing 10% FBS, and cells were incubated for an additional 30 min at 37 °C in the dark. Nuclei were counterstained with DAPI for 2 min, and the cells were washed twice with PBS. Fluorescence images were captured using a Nikon A1R confocal microscope.

### Gene Expression Omnibus (GEO) data analysis

Gene expression analysis was performed using the GEO dataset GSE218702, which includes proteomic profiles from wild-type (WT) mice at 0, 3, 7, and 14 days post-sciatic nerve injury. Data were retrieved using the GEO query package in R and normalized using the Robust Multi-array Average (RMA) method. Differential expression analysis was conducted using the *limma* package with a significance threshold of *p* < 0.05 and fold change > 2. Differentially expressed genes (DEGs) were visualized using volcano plots, heatmaps, fuzzy clustering, and pathway enrichment analyses. All visualizations were generated using the *ggplot2* package in R.

### Protein-Protein Interaction (PPI) network analysis

Protein-protein interaction analysis was performed to investigate the interaction between ANT2 and mitophagy-related proteins using the STRING database (v11.5, https://string-db.org). A minimum required interaction score of 0.7 (high confidence) was applied. Interaction networks were visualized using Cytoscape software (v3.9.1).

### MitoCarta database cross-analysis

Mitochondrial-associated genes were retrieved from the MitoCarta3.0 database. Differentially expressed proteins identified by TMT-based proteomics and genes enriched in key pathways were intersected with the MitoCarta gene set using Venn diagram analysis in R. Overlapping genes were identified as potential mitochondria-related targets.

### 3D surface plot of calcium imaging

Three-dimensional surface plots of Fluo-4 fluorescence intensity were generated using the Interactive 3D Surface Plot plugin in ImageJ. The Z-axis represents the relative fluorescence intensity of [Ca^2+^]ᵢ, providing a visual representation of calcium signal dynamics across time and spatial distribution.

### Protein digestion and TMT labeling

Samples were rinsed with PBS and subjected to proteolytic digestion using sequencing-grade trypsin and protease in Eppendorf tubes. After digestion, peptides were transferred to clean 1.5 mL tubes for pH verification and desalted using SOLAμ columns (Thermo Fisher Scientific). Peptides were then labeled using the TMTpro 16-plex Isobaric Label Reagent Set (Thermo Scientific) following the manufacturer's protocol. Labeled peptides were separated by high-performance liquid chromatography (HPLC) and pooled into 30 fractions for downstream LC-MS/MS analysis.

### LC-MS/MS analysis

LC-MS/MS analysis was conducted using a DIONEX UltiMate 3000 RSLCnano system coupled with an Orbitrap Exploris 480 mass spectrometer (Thermo Scientific) equipped with a FAIMS Pro interface. Data were acquired in data-dependent acquisition (DDA) mode. High-resolution full MS scans and MS/MS spectra were used for peptide identification and quantification.

### Gene Set Enrichment Analysis (GSEA)

GSEA was performed using GSEA software (v4.3.2, Broad Institute). Proteins identified from TMT-based proteomics were ranked based on log_2_ fold change and *p*-value. Gene sets for oxidative phosphorylation and calcium signaling pathways were retrieved from the Molecular Signatures Database (MSigDB v7.4). Enrichment scores, normalized enrichment scores (NES), and false discovery rate (FDR) *q*-values were calculated to evaluate pathway significance.

### GO and KEGG pathway enrichment analysis

Differentially expressed proteins were analyzed for Gene Ontology (GO) terms and Kyoto Encyclopedia of Genes and Genomes (KEGG) pathways using the *clusterProfiler* package (v4.4.4) in R. Functional terms with an adjusted *p*-value < 0.05 were considered significantly enriched.

### Molecular docking analysis

Molecular docking was performed using Schrödinger software. The receptor protein was prepared with the Protein Preparation Wizard, including residue optimization, hydrogen bond assignment, solvent removal, and energy minimization. Ligands were processed using LigPrep with the OPLS3e force field. Docking was performed using Glide in standard precision (SP) mode, based on the prepared grid and ligand files. Binding poses and interactions were visualized using PyMOL 2.6.

### Statistical analysis

All data are presented as mean ± standard deviation (SD). Sample size was determined based on prior experiments with similar methodologies. Statistical analyses were performed using GraphPad Prism 10.0. Comparisons between groups were assessed using two-tailed *t*-tests, one-way ANOVA, or two-way ANOVA as appropriate. Statistical significance was defined as *p* < 0.05. Asterisks denote significance as follows: ns (not significant), ^*^p < 0.05, ^**^p < 0.01, ^***^p < 0.001, and ^****^p < 0.0001.

## Results

### Moderate elevation of Ca^2+^ promotes Schwann cell functions after peripheral nerve injury

**Figure 1:**
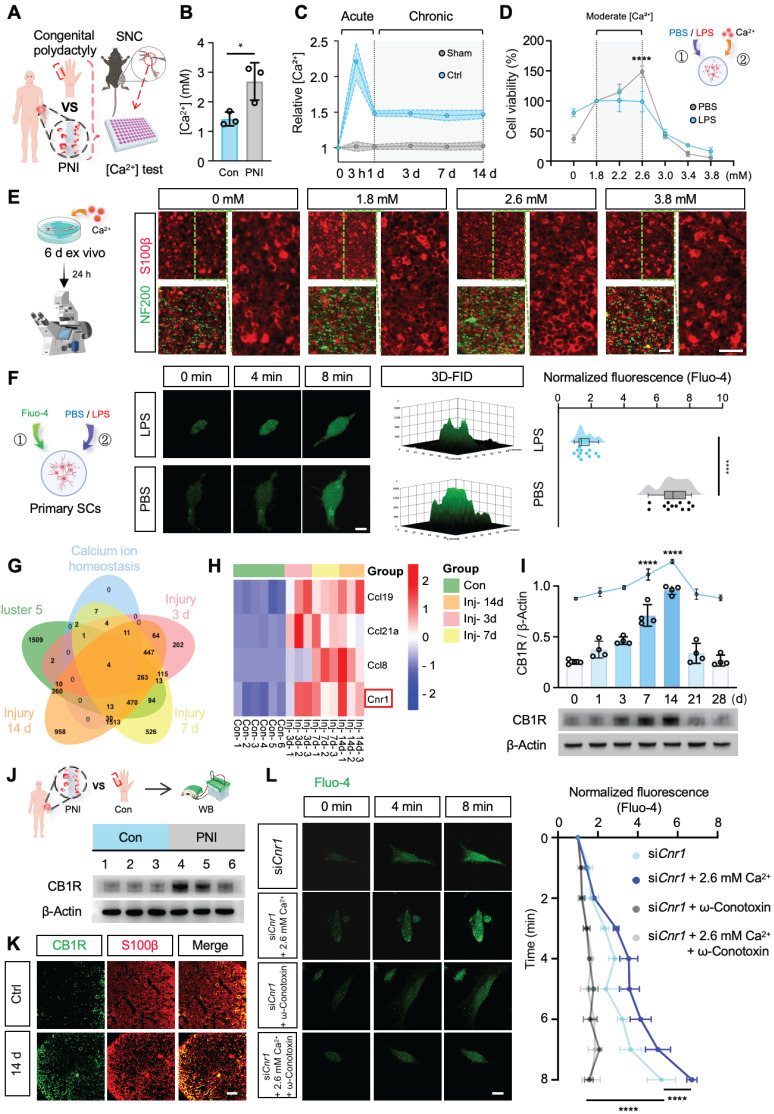
** Moderate extracellular calcium promotes Schwann cell survival via CB1R-mediated Ca^2+^ influx. (A)** Schematic illustration: Sciatic nerve tissues excised intraoperatively from patients with severe peripheral nerve injury (PNI group) and digital nerves from patients with congenital polydactyly (Con group) were collected. A mouse sciatic nerve crush model was also established. All samples were used for calcium concentration analysis. **(B)** [Ca^2+^] in sciatic nerves collected 6 h after injury compared with control (n = 3). **(C)** Relative Ca^2+^ levels in injured and distal sciatic nerves at 3 h, 1 d, 3 d, 7 d, and 14 d post-injury (n = 5). **(D)** Cell viability of primary Schwann cells (SCs) cultured under graded extracellular Ca^2+^ concentrations in PBS- or LPS-treated conditions (n = 3). **(E)** Representative IF images of *ex vivo*-cultured sciatic nerves treated with indicated Ca^2+^ concentrations. Myelin labeled with S100β (red), axons with NF200 (green). Scale bar = 20 μm. **(F)** Intracellular Ca^2+^ levels in primary SCs stimulated with 2.6 mM Ca^2+^ under LPS or PBS treatment, measured by Fluo-4 fluorescence at 0, 4, and 8 min. Right: 3D surface plot and quantification of fluorescence intensity at 8 min (n = 12 cells/group). Scale bar = 10 μm. **(G)** Venn diagram of DEGs at 3, 7, and 14 d post-injury intersected with calcium homeostasis-related genes. **(H)** Heatmap showing expression of selected DEGs (*Ccl19, Ccl21a, Ccl8, Cnr1*) across time points from GSE218702. **(I)** Western blot analysis of CB1R expression at indicated time points post-injury. Upper: quantification of CB1R/β-Actin; lower: representative blot (n = 4). **(J)** Western blot analysis of CB1R protein expression in human nerve tissues before and after injury. Top: Schematic illustration; bottom: representative blots (n = 3). **(K)** IF staining of CB1R (green) and S100β (red) in sciatic nerves at baseline and 14 d post-injury. Scale bar = 20 μm. **(L)** Time-lapse Fluo-4 imaging of Ca^2+^ influx in CB1R-knockdown SCs (si*Cnr1*) treated with 2.6 mM Ca^2+^ or ω-conotoxin. Right: normalized fluorescence intensity quantification (n = 3). Scale bar = 10 μm. All data are presented as mean ± SD. Statistical tests: unpaired two-tailed t-test (B, C, F); one-way ANOVA with Tukey's post hoc test (I); two-way ANOVA with Bonferroni's post hoc test (D, L). ^*^p < 0.05, ^****^p < 0.0001. CB1R, cannabinoid receptor 1; SCs, Schwann cells; PNI, peripheral nerve injury; SNC, sciatic nerve crush.

To investigate the role of calcium ions (Ca^2+^) in the process of nerve regeneration, we first determined the dynamic changes of Ca^2+^ in the microenvironment following nerve injury. Peripheral nerve samples were collected intraoperatively from patients with acute peripheral nerve injury (≤ 6 h post-injury) and from nerves excised during polydactyly correction surgery (Figure 1A, Table S1). Calcium levels were measured using a colorimetric assay kit, and the results revealed a significant increase (~1.7-fold) in Ca^2+^ concentration in the injured nerve tissues (Figure 1B). Due to the limited availability of clinical samples and the difficulty in dynamically monitoring calcium fluctuations, we established a mouse sciatic nerve crush injury model (Figure S1A) to systematically investigate the temporal changes of Ca^2+^ during the early stages of nerve injury. Using a calcium colorimetric assay kit, we measured extracellular free Ca^2+^ levels at various time points (3 h, 1 d, 3 d, 7 d, and 14 d) within the injury site and distal nerve segments (Figure 1A). The results showed a rapid increase in tissue Ca^2+^ concentration immediately post-injury, peaking at 3 h and then stabilizing from day 1 to day 14 (Figure 1C). Considering the critical role of Schwann cells (SCs) in peripheral nerve regeneration, we further explored the effects of increased extracellular Ca^2+^ on SC functions following injury. Primary SCs were isolated from the sciatic nerves of 2-day-old rats (Figure S1B) and treated with either PBS or lipopolysaccharide (LPS), the latter known to induce diverse cellular responses in SCs 31. Different concentrations of CaCl_2_ were added to the base medium (DMEM-F12, containing 1.8 mM Ca^2+^) to simulate variations in extracellular Ca^2+^ post-injury. SC viability under these conditions was assessed using the CCK-8 assay (Figure 1D). The results revealed that mildly increased extracellular Ca^2+^ significantly promoted SC proliferation in the PBS-treated group, while no noticeable changes in proliferation were observed in the LPS-treated group. Additionally, environments with extremely low or excessively high Ca^2+^ levels induced SC apoptosis. Sciatic nerve explant assays, utilizing immunofluorescent staining for myelin (S100β) and axons (NF200), further demonstrated that a moderate elevation of extracellular Ca^2+^ notably reduced abnormal myelin (Figure 1E and Figure S1C). These findings suggest that moderate increases in extracellular Ca^2+^ enhance SC proliferation and protect myelin integrity.

To clarify whether variations in extracellular Ca^2+^ influence intracellular Ca^2+^ levels ([Ca^2+^]_i_) in SCs, we cultured cells in a medium containing 2.6 mM Ca^2+^ and measured intracellular Ca^2+^ levels using the Fluo-4 calcium probe. Elevated extracellular Ca^2+^ significantly increased intracellular Ca^2+^ under physiological conditions; however, intracellular Ca^2+^ levels in pathological SCs showed no significant changes (Figure 1F). Thus, we hypothesize that nerve injury may activate specific proteins or signaling pathways that impede extracellular Ca^2+^ influx, suppressing SC proliferation and impairing regeneration.

### Inhibition of CB1R promotes intracellular Ca^2+^ influx in SCs

To identify upstream regulators responsible for Ca^2+^ fluctuations following peripheral nerve injury, we utilized the GEO database (GSE218702) to explore associations between injury-induced gene expression and Ca^2+^ signaling through systematic bioinformatic analysis. Differentially expressed genes were initially identified by comparing expression profiles at 3, 7, and 14 days post-injury with baseline (0 d) levels (Figure S1D). Subsequent clustering analysis using Mfuzz software categorized these genes into clusters 1-6 (Figure S1E). Cluster 5 was selected for detailed analysis due to its variation, which highly correlated with Ca^2+^ dynamics after injury. Venn diagram analysis identified intersections among the differentially expressed genes, cluster 5, and genes enriched in GO terms related to Ca^2+^ regulation, highlighting *Ccl19, Ccl21a, Ccl8,* and *Cnr1* as key candidate genes (Figure 1G-H). The *Ccl* family genes were primarily expressed in macrophages, whereas *Cnr1* emerged as the core target gene for subsequent investigation.

*Cnr1* encodes cannabinoid receptor 1 (CB1R), a G-protein-coupled receptor intimately involved in Ca^2+^ signaling and known for its diverse roles in neurobiology. However, its function in peripheral nerve injury remains to be fully understood. Western blot analysis validated the dynamic expression pattern of CB1R, showing an early increase at day 1, with peak expression at days 7 and 14 post-injury (Figure 1I), consistent with predictions from the GEO database. Meanwhile, Western blot analysis of previously collected human nerve samples (Figure 1A) also revealed a remarkable upregulation of CB1R following nerve injury (Figure 1J). Immunofluorescence staining confirmed that CB1R was predominantly expressed in SCs, with a significant increase at 14 days post-injury (Figure 1K). Consequently, we propose that CB1R is a crucial upstream regulator of Ca^2+^ fluctuations following peripheral nerve injury.

CB1R-mediated Ca^2+^ regulation exhibits significant cell-type specificity, potentially due to distinct downstream pathways and ion-channel expression profiles 18-21. To investigate the specific role of CB1R in Ca^2+^ regulation in SCs, we reduced *Cnr1* expression in SCs using siRNA (si*Cnr1*). Fluo-4-based Ca^2+^ imaging revealed that intracellular Ca^2+^ levels in the si*Cnr1* group were significantly elevated compared to controls, especially under high extracellular Ca^2+^ conditions (2.6 mM) (Figure S1F-G). Previous studies suggest that CB1R can reduce Ca^2+^ influx by inhibiting voltage-gated calcium channels 32,33. Therefore, we treated SCs with voltage-gated calcium channel inhibitors (ω-Conotoxin GVIA/MVIIC, targeting L-, N-, and P/Q-type channels). The results demonstrated that the administration of these inhibitors reversed the increase in intracellular Ca^2+^ levels induced by *Cnr1* silencing, regardless of extracellular Ca^2+^ supplementation (Figure 1L). These findings indicate that CB1R inhibition promotes Ca^2+^ influx via voltage-gated calcium channels in injured SCs.

### CB1R knockout promotes peripheral nerve regeneration without affecting environmental calcium levels post-injury

**Figure 2:**
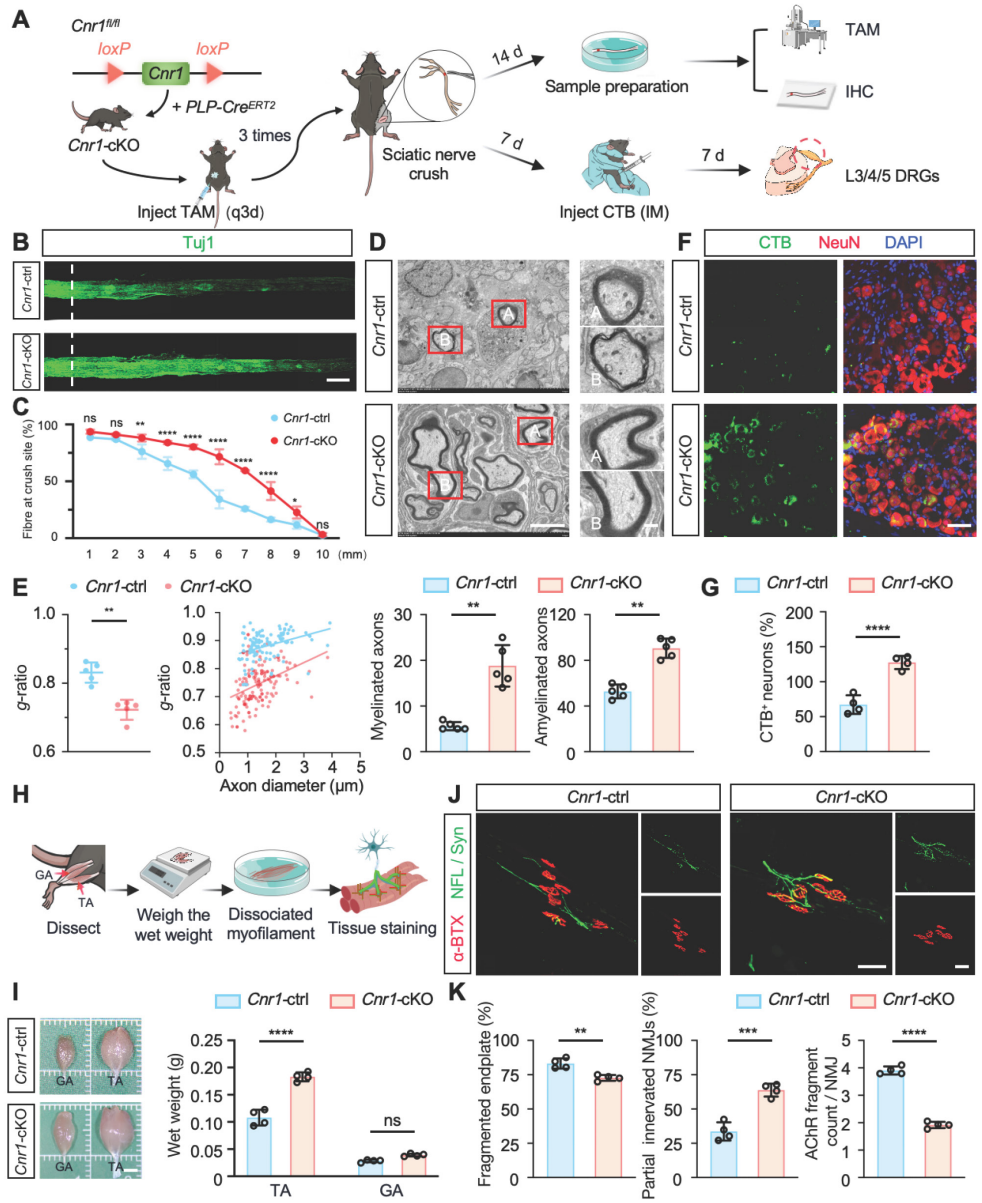
** Schwann cell-specific deletion of *Cnr1* enhances axonal regeneration, remyelination, and neuromuscular reinnervation. (A)** Schematic of TAM-induced SC-specific *Cnr1* knockout mice (*Cnr1-*cKO*, Cnr1^fl/fl^; PLP-Cre^ERT2^*) and experimental timeline. **(B)** Representative IF images of regenerating axons in the distal sciatic nerve labeled with Tuj1 (green). Scale bar = 1 mm. n = 3 mice. **(C)** Quantification of axon regeneration at 3-9 mm distal to the injury site. n = 3 mice. **(D)** TEM images of remyelinated axons in the distal stump. Red boxes indicate magnified regions. Scale bars: 5 μm (overview), 2 μm (zoomed). **(E)** Quantification of myelin* g*-ratio (left), number of myelinated axons (middle), and amyelinated axons (right) in *Cnr1*-cKO and control groups. n = 5 mice. **(F)** Representative IF staining of DRG neurons labeled with NeuN (red), CTB-traced neurons (green), and DAPI (blue). Scale bar = 100 μm. **(G)** Quantification of CTB-positive DRG neurons showing a significant increase in the *Cnr1*-cKO group. n = 4 mice. **(H)** Schematic workflow of muscle analysis at 14 days post-injury. **(I)** Quantification of wet weight of TA and GA muscles on the injured side. TA muscle weight was significantly increased in the *Cnr1*-cKO group, while GA showed no significant difference. n = 4 mice. **(J)** Representative IF images of NMJs labeled with α-BTX (red), NFL/Syn (green). Scale bar = 100 μm. **(K)** Quantification of NMJ morphology, showing a reduced proportion of fragmented endplates (left), decreased partially innervated NMJs (middle), and enhanced AChR clustering (right) in the *Cnr1*-cKO group. n = 4 mice. All data are presented as mean ± SD. Statistical analysis: two-way ANOVA with Bonferroni's post hoc test (C), bivariate linear regression and one-way ANOVA with Tukey's post hoc test (E, I), unpaired two-tailed t-test (G, K). ^*^p < 0.05, ^**^p < 0.01, ^***^p < 0.001, ^****^p < 0.0001.; ns, not significant. TAM, tamoxifen; TEM, transmission electron microscopy; IM, Intramuscular injection; IHC, Immunohistochemistry; CTB, Cholera Toxin Subunit B; DRG, Dorsal Root Ganglion; TA,tibialis anterior; GA, gastrocnemius; NMJs, neuromuscular junctions; α-BTX, acetylcholine receptors; NFL/Syn, neurofilament/synaptic vesicles.

Next, we further elucidated the biological role of CB1R in peripheral nerve regeneration by generating tamoxifen (TAM)-inducible Schwann cell-specific *Cnr1* knockout mice (*Cnr1^fl/fl^; PLP-Cre^ERT2^*, *Cnr1-*cKO) and establishing a sciatic nerve crush injury model (Figure 2A). Immunofluorescent staining for axons (Tuj1) revealed that at 14 days post-injury, *Cnr1-*cKO mice exhibited significantly increased axonal regeneration in distal segments (3-9 mm from the injury site) compared to control mice (Figure 2B-C). Further ultrastructural analyses using transmission electron microscopy (TEM) demonstrated that the *Cnr1-*cKO mice displayed significantly improved remyelination and an increased proportion of myelinated axons (Figure 2D-E). Additionally, neuronal tracing using cholera toxin subunit B (CTB), injected at 7 days post-injury, revealed a significantly higher number of labeled neurons in the dorsal root ganglia (DRG) of *Cnr1-*cKO mice at 14 days post-injury compared to controls (Figure 2F-G). These findings indicate that CB1R knockout significantly facilitates axonal regeneration and remyelination following peripheral nerve injury.

Functional recovery after peripheral nerve injury critically depends on re-establishing neuromuscular junctions (NMJs) and reinnervating target muscles 34. To further assess this, we examined the wet weights of the tibialis anterior (TA) and gastrocnemius (GA) muscles at 14 days post-injury (Figure 2H). *Cnr1-*cKO mice exhibited a significantly increased wet weight of the tibialis anterior compared to controls, whereas no significant differences were observed in the gastrocnemius muscle (Figure 2I). Morphological analyses of NMJs using immunofluorescence staining for acetylcholine receptors (α-BTX), neurofilament (NFL), and synaptic vesicle protein (Syn) revealed that *Cnr1-*cKO mice had significantly lower proportions of fragmented NMJs, increased numbers of partially innervated NMJs, and improved AChR clustering (Figure 2J-K). These results demonstrate that CB1R knockout accelerates muscle functional recovery.

**Figure 3:**
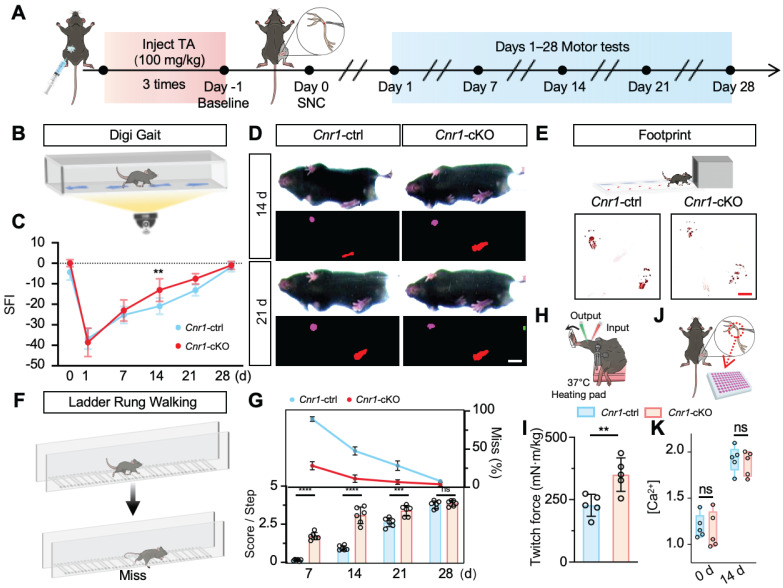
** Schwann cell-specific *Cnr1* deletion enhances functional recovery following sciatic nerve injury. (A)** Schematic of experimental workflow for functional assessment in *Cnr1*-cKO and control mice. **(B)** Representative image of the DigiGait system used to evaluate gait performance post-injury. **(C)** Quantification of SFI showing significantly improved motor recovery in the *Cnr1*-cKO group at 14 and 21 days post-injury. n = 6 mice (control), n = 5 mice (*Cnr1*-cKO). **(D)** Representative gait heatmaps from DigiGait analysis showing improved paw placement and stride in *Cnr1*-cKO mice. Scale bar = 1 cm. **(E)** Schematic and representative footprint images demonstrating increased plantar contact area in *Cnr1*-cKO mice at 14 days post-injury. Scale bar = 1 cm. **(F)** Schematic of the ladder rung walking test used to assess fine motor coordination. **(G)** Quantification of ladder rung walking performance showing significantly higher scores in *Cnr1*-cKO mice at 7, 14, and 21 days post-injury. n = 6 mice. **(H)** Schematic of muscle force testing setup for hindlimb muscle strength evaluation. **(I)** Quantification of twitch force at 14 days post-injury, showing enhanced muscle contractile strength in the *Cnr1*-cKO group. n = 5 mice. **(J)** Diagram showing sciatic nerve tissue collection from *Cnr1*-ctrl and *Cnr1*-cKO mice for calcium concentration analysis. **(K)** Extracellular calcium concentrations in sciatic nerves of *Cnr1*-ctrl and *Cnr1*-cKO mice at day 0 and 14 post-injury (n = 5). All data are presented as mean ± SD. Statistical analysis: two-way ANOVA with Bonferroni's post hoc test (C, G, K), unpaired two-tailed t-test (I). ^**^p < 0.01, ^***^p < 0.001, ^****^p < 0.0001; ns, not significant. SFI, sciatic functional index; TA,tibialis anterior; SNC, sciatic nerve crush.

To correlate morphological improvements with functional outcomes, we conducted comprehensive behavioral assessments, including DigiGait analysis, footprint analysis, the sciatic functional index (SFI), and horizontal ladder rung tests (Figure 3A). Results consistently showed that motor performance (SFI scores, paw print area, slip rate, and walking scores) was significantly enhanced in *Cnr1-*cKO mice compared to control mice at both 14 and 21 days post-injury (Figure 3B-G). Moreover, direct muscle contractile force measurements confirmed that muscle strength recovered significantly better in the *Cnr1-*cKO group at 14 days post-injury (Figure 3H-I). We measured the tissue Ca^2+^ concentration in *Cnr1-*cKO mice before injury and at 14 days post-injury. The results showed that *Cnr1* deletion did not affect the local Ca^2+^ concentration in the microenvironment after nerve injury (Figure 3J-K). These findings strongly suggest that Schwann cell-specific deletion of CB1R facilitates axonal regeneration and remyelination and markedly enhances functional recovery following peripheral nerve injury.

### ANT2 was upregulated by CB1R knockout-mediated calcium influx

**Figure 4:**
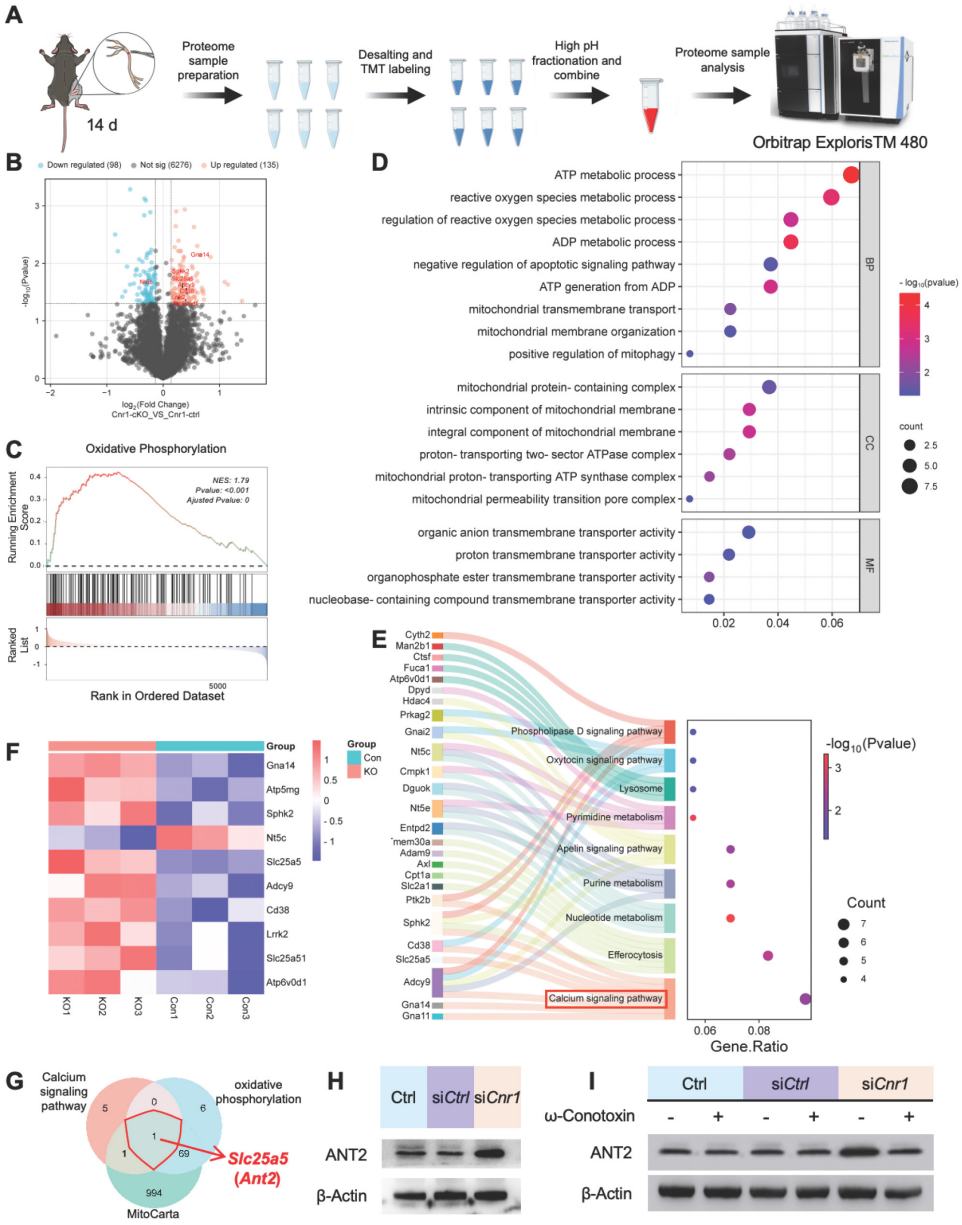
Proteomic analysis of CB1R-mediated Ca^2+^ regulation and its downstream pathways. **(A)** Schematic diagram of the proteomic analysis. **(B)** Volcano plot showing differentially expressed proteins between *Cnr1*-cKO and *Cnr1*-ctrl groups with 135 upregulated and 98 downregulated. **(C)** Gene Set Enrichment Analysis (GSEA) indicating significant upregulation of the oxidative phosphorylation pathway in the *Cnr1*-cKO group. Normalized enrichment score (NES) = 1.79, ^**^p < 0.001. **(D)** Gene Ontology (GO) enrichment analysis showing that differentially expressed proteins are primarily involved in ATP metabolism, ROS regulation, mitochondrial transmembrane transport, and other mitochondrial-related biological processes. **(E)** Kyoto Encyclopedia of Genes and Genomes (KEGG) pathway analysis highlighting signaling pathways associated with nerve repair, with significant enrichment in the calcium signaling pathway (highlighted in red). **(F)** Heatmap displaying expression levels of key differentially expressed proteins (including *Slc25a5*, *Gna14*, and *Atp5mg*) between *Cnr1*-cKO and *Cnr1*-ctrl mice at 14 days post-injury. **(G)** Intersection analysis of genes enriched in the GSEA calcium signaling pathway and the KEGG oxidative phosphorylation pathway with the mitochondrial gene database (MitoCarta) identified *Slc25a5* (*Ant2*) as the only overlapping gene. **(H)** Western blot analyzed the expression of ANT2 after CB1R knockdown (si*Cnr1*). **(I)** Western blot analysis of ANT2 expression with or without ω-Conotoxin administration. ANT2, adenine nucleotide translocase 2.

To further explore the molecular mechanisms underlying CB1R-mediated nerve regeneration, we performed TMT-based proteomic analysis on sciatic nerve tissues from *Cnr1-*cKO and control mice (*Cnr1-*ctrl) at 14 days post-injury (Figure 4A). A total of 233 differentially expressed proteins were identified, comprising 135 upregulated and 98 downregulated proteins (Figure 4B). Gene Set Enrichment Analysis (GSEA) demonstrated significant upregulation of the oxidative phosphorylation pathway following CB1R knockout (Figure 4C), indicating enhanced energy metabolism in nerve tissues to support regenerative processes. Gene Ontology (GO) enrichment analysis further revealed that differentially expressed proteins were predominantly associated with energy metabolism and mitochondrial function (Figure 4D), highlighting mitochondria as potential central players in CB1R-mediated nerve regeneration. Additionally, KEGG pathway analysis underscored the importance of the calcium signaling pathway (Figure 4E), consistent with previous GEO database analyses, thereby reinforcing Ca^2+^ as a crucial regulator in peripheral nerve regeneration.

To elucidate the key role of CB1R in Ca^2+^-mediated mitochondrial remodeling, we conducted an integrated analysis of the differentially expressed proteins, ultimately identifying ten candidate proteins, including *Slc25a5 (Ant2)*, *Gna14*, and *Atp5mg*, which are closely related to mitochondrial function. Heatmap analysis displayed their dynamic expression changes (Figure 4F). Focusing on genes that regulate mitochondrial quality and quantity, we intersected genes enriched in the GSEA calcium signaling pathway and KEGG oxidative phosphorylation pathway with mitochondrial-associated genes, ultimately identifying *Slc25a5 (Ant2)* as the sole intersectional gene (Figure 4G).

ANT2, an essential ADP/ATP translocator in the mitochondrial membrane, plays a key role in mitochondrial energy metabolism and functional integrity 35,36. To validate the regulation of ANT2 expression by CB1R via Ca^2+^ signaling, we performed Western blot analysis. The results showed that silencing CB1R significantly upregulated ANT2 expression—an effect that was effectively reversed by the calcium channel inhibitor ω-Conotoxin (Figure 4H-I and Figure S2A-B). These results collectively demonstrate that CB1R regulates mitochondrial functional remodeling through intracellular Ca^2+^ signaling pathways, as well as modulating ANT2 expression.

### CB1R promotes Schwann cell mitophagy via the Ca^2+^-ANT2-PINK1/Parkin axis

**Figure 5:**
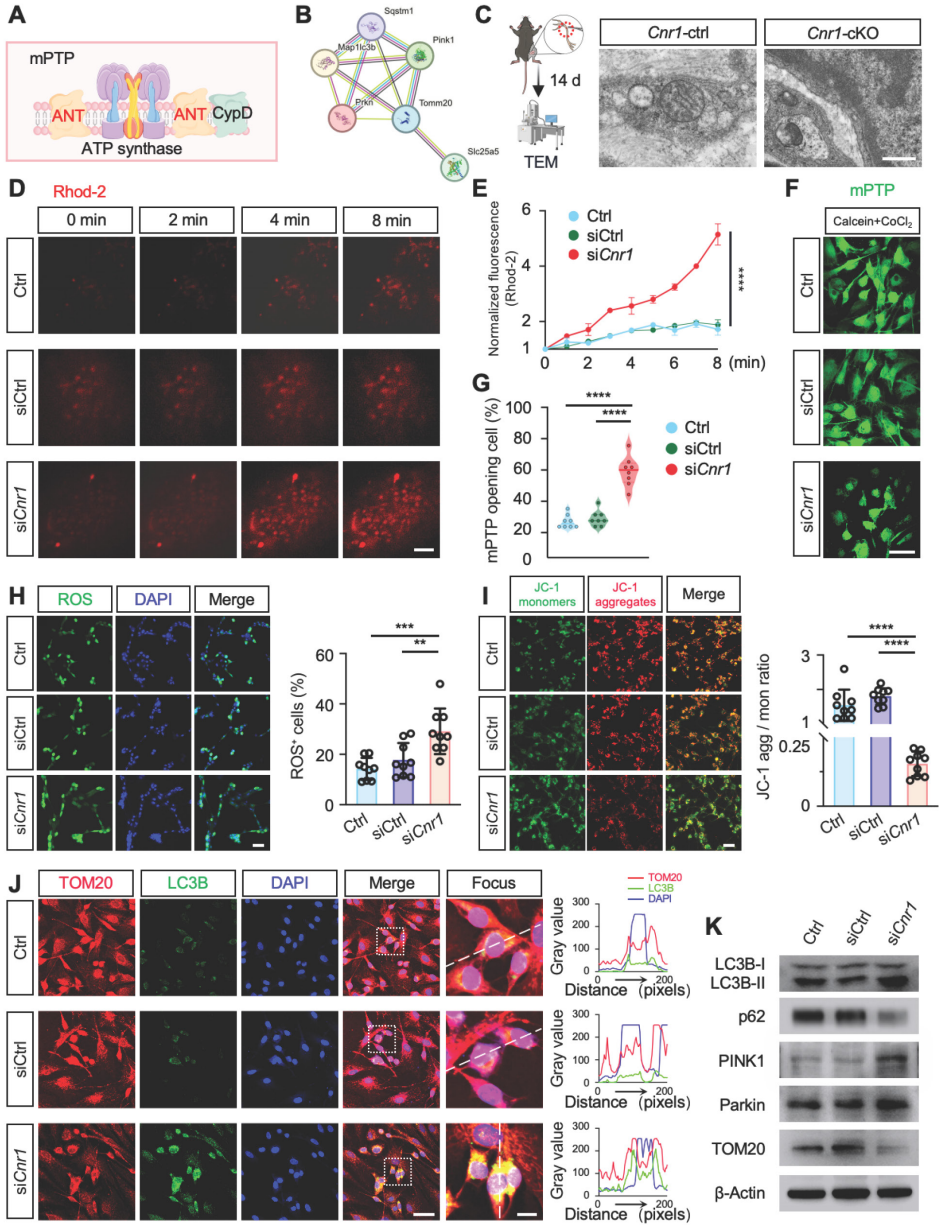
** Inhibition of CB1R enhances mitophagy via activation of the PINK1/Parkin pathway. (A)** Schematic illustration of ANT2-mediated regulation of mPTP opening and its role in mitophagy. **(B)** STRING database prediction of protein-protein interactions between ANT2 and components of the PINK1/Parkin mitophagy pathway. **(C)** TEM images of sciatic nerve tissues from *Cnr1*-ctrl and *Cnr1*-cKO mice showing improved mitochondrial ultrastructure in the *Cnr1*-cKO group. Scale bar = 500 nm. **(D)** Representative Rhod-2 fluorescence images of mitochondrial Ca^2+^ dynamics at 0, 2, 4, and 8 min in Ctrl, siCtrl, and si*Cnr1* groups. Scale bar = 10 μm. **(E)** Quantification of Rhod-2 fluorescence intensity over time. n = 3 replicates. **(F)** Live-cell imaging of mPTP opening using the Calcein-CoCl_2_ assay. Green fluorescence indicates mPTP state. Scale bar = 20 μm. **(G)** Quantification of mPTP-positive cells. n = 8 images from 3 independent experiments. **(H)** Representative ROS fluorescence staining showing intracellular ROS levels in Ctrl, siCtrl, and si*Cnr1* groups. Scale bar = 20 μm. Quantification of ROS⁺ cells. n = 9 images from 3 replicates. **(I)** JC-1 assay of mitochondrial membrane potential and quantification of JC-1 aggregate/monomer ratio in the three groups. Scale bar = 20 μm. n = 9 images from 3 replicates. **(J)** Representative IF staining of LC3B (green, autophagy marker) and TOM20 (red, mitochondrial marker). Scale bars = 20 μm (overview), 5 μm (zoomed region). **(K)** Western blot analysis of mitophagy-related proteins. The si*Cnr1* group showed increased levels of LC3B-II/I, PINK1, and Parkin, and decreased expression of TOM20 and p62. All data are presented as mean ± SD. Statistical analysis: two-way ANOVA with Bonferroni's post hoc test (E), one-way ANOVA with Tukey's post hoc test (G, H, I). ^**^p < 0.01, ^***^p < 0.001, ^****^p < 0.0001. mPTP, mitochondrial permeability transition pore; TEM, transmission electron microscopy; ROS, reactive oxygen species; JC-1, 5,5′,6,6′-tetrachloro-1,1′,3,3′-tetraethylbenzimidazolylcarbocyanine iodide.

ANT2 is an essential component of the mitochondrial permeability transition pore (mPTP), located in the mitochondrial inner membrane, where it regulates pore opening 36,37 (Figure 5A). STRING database analysis further indicated a potential functional interaction between ANT2 and the PINK1-Parkin mitophagy pathway (Figure 5B). To verify this relationship, Western blot analysis was performed, revealing that silencing ANT2 significantly reduced the expression of Parkin, a critical mitophagy protein, while markedly increasing the expression of the autophagy substrate p62, the mitochondrial marker TOM20, and the LC3BII/I ratio (Figure S2C-E). These results indicate that ANT2 suppression inhibits mitophagy. Accordingly, improved mitochondrial structure and increased mitophagy were observed in *Cnr1-*cKO mice, whose ANT2 was significantly upregulated in sciatic nerve (Figure 5C).

A reduction in mitochondrial membrane potential (ΔΨm) is a key trigger for mitophagy 38-40, achieved by mPTP opening during mitochondrial Ca^2+^ overload or ROS accumulation 41,42. Therefore, we performed real-time fluorescence imaging using the mitochondria-specific calcium probe Rhod-2, assessed mPTP opening through live-cell fluorescence assays, and conducted JC-1 and ROS staining analyses (Figure 5D-I). The results showed that CB1R silencing significantly enhanced mPTP opening, decreased ΔΨm, and was accompanied by an increase in ROS levels (Figure 5D-I). These results suggested that silencing CB1R promoted mitophagy by reducing ΔΨm.

Immunofluorescence analysis further confirmed increased LC3B fluorescence intensity and positive puncta, as well as enhanced colocalization with the mitochondrial marker TOM20 in Schwann cells following CB1R silencing, indicating heightened mitophagic activity (Figure 5J). Western blot analysis supported these observations, showing significant upregulation of mitophagy-associated proteins LC3BII/I, PINK1, and Parkin, accompanied by reduced expression of TOM20 and p62 (Figure 5K and Figure S2F). Intervention with the calcium channel inhibitor ω-Conotoxin notably reversed the enhanced mitophagy induced by CB1R inhibition (Figure S2G-I). Collectively, these results confirm that silencing CB1R in pathological SCs enhances mitochondrial function by promoting mitophagy via the Ca^2+^-dependent PINK1/Parkin pathway.

### CB1R-regulated mitophagy facilitates peripheral nerve regeneration

**Figure 6:**
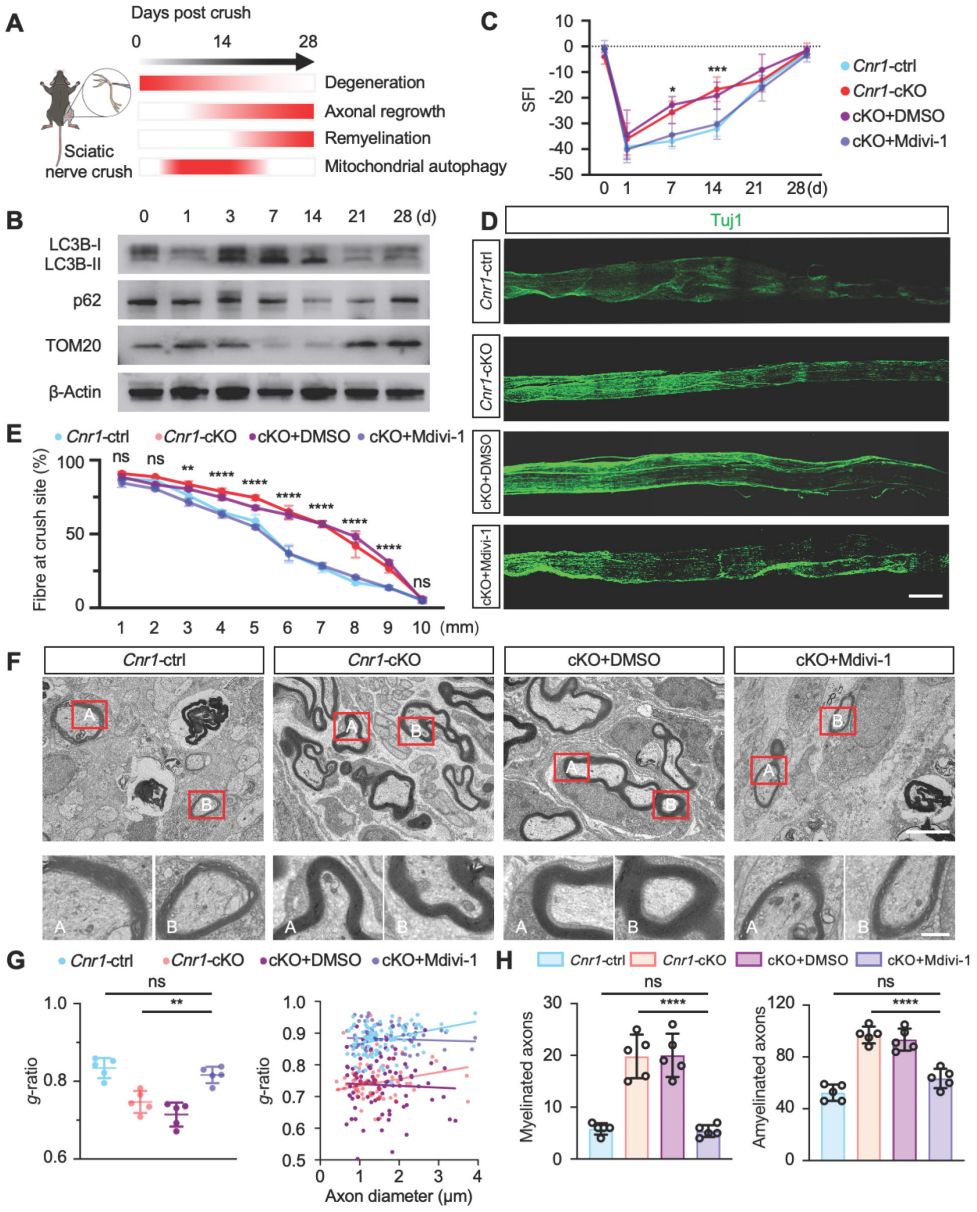
** CB1R promotes peripheral nerve repair by regulating mitophagy. (A)** Schematic illustration of the dynamic repair process following sciatic nerve injury over a 28-day period. Myelin degradation initiates immediately post-injury and peaks at 3 days, axonal regeneration begins around day 7 and peaks at day 14, while mitophagy is first observed at day 3 and markedly increases between days 7 and 14. **(B)** Western blot analysis of mitophagy-related proteins (LC3B-I/II, p62, and TOM20) at 0, 3, 7, 14, and 28 days post-injury, showing a significant upregulation of mitophagy markers between days 7 and 14. n = 4 mice. **(C)** Quantification of SFI scores in *Cnr1*-ctrl and *Cnr1*-cKO mice, with or without Mdivi-1 treatment, at 14 and 21 days post-injury. n = 6 mice per group. **(D)** Representative IF images of regenerating axons in the distal sciatic nerve labeled with Tuj1 (green). Scale bar = 1 mm. **(E)** Quantification of axon regeneration at 3-9 mm distal to the injury site across indicated groups. n = 3 mice. **(F)** TEM images of axonal remyelination in the distal sciatic nerve. Red boxes indicate magnified regions. Scale bars: 5 μm (overview), 1 μm (zoomed). **(G)** Quantification of myelin g-ratio showing reduced values in *Cnr1*-cKO mice, which were reversed by Mdivi-1 treatment. Right: scatter plot displaying the correlation between g-ratio and axon diameter. n = 5 mice. **(H)** Quantification of the number of myelinated axons (left) and amyelinated axons (right). n = 5 mice. All data are presented as mean ± SD. Statistical analysis: two-way ANOVA with Bonferroni's post hoc test (C, E), bivariate linear regression and one-way ANOVA with Tukey's post hoc test (G), one-way ANOVA with Tukey's post hoc test (H). ^*^p < 0.05, ^**^p < 0.01, ^***^p < 0.001, ^****^p < 0.0001.; ns, not significant. SFI, sciatic functional index; Mdivi-1, mitochondrial division inhibitor-1.

During the 28-day recovery period following peripheral nerve injury, myelin degradation begins immediately post-injury, peaking at day 3, while axonal regeneration gradually initiates from day 7 and reaches its peak by day 14 (Figure 6A). Western blot analysis further demonstrated that mitophagy activation begins at day 3 post-injury, with significant enhancement observed between days 7 and 14 (Figure 6B and Figure S3A). This temporal correlation between mitophagy activation and the critical periods of axonal regeneration and myelin remodeling suggests that mitophagy plays a key role in nerve repair. Previous studies from our group indicated that mitophagy mediated by the PINK1-Parkin pathway promotes nerve repair post-injury 43, yet the detailed mechanisms remained incompletely defined.

To elucidate the specific role of CB1R-mediated mitophagy in peripheral nerve regeneration, mice with peripheral nerve injuries were treated daily with the mitophagy inhibitor Mdivi-1 (40 mg/kg, intraperitoneally). This inhibitor reduces mitochondrial fission by suppressing Drp1 GTPase activity, thereby inhibiting mitophagy 44,45. Functional assessments using DigiGait analysis and the sciatic functional index (SFI) scoring demonstrated that Mdivi-1 significantly reversed the improved functional recovery observed in *Cnr1-*cKO mice, resulting in no significant difference compared with the control group (*Cnr1-*ctrl) (Figure 6C). Immunofluorescence staining of axons (Tuj1) showed that the enhanced axonal regeneration observed in *Cnr1-*cKO mice was abolished by Mdivi-1 treatment (Figure 6D-E). Transmission electron microscopy further confirmed that Mdivi-1 reversed the beneficial effects of CB1R knockout on remyelination and the proportion of myelinated axons, resulting in levels comparable to those of the control group (Figure 6F-H). Collectively, these findings demonstrate that inhibition of mitophagy with Mdivi-1 effectively negates the pro-regenerative effects of CB1R knockout.

### JD5037 promotes nerve regeneration

**Figure 7:**
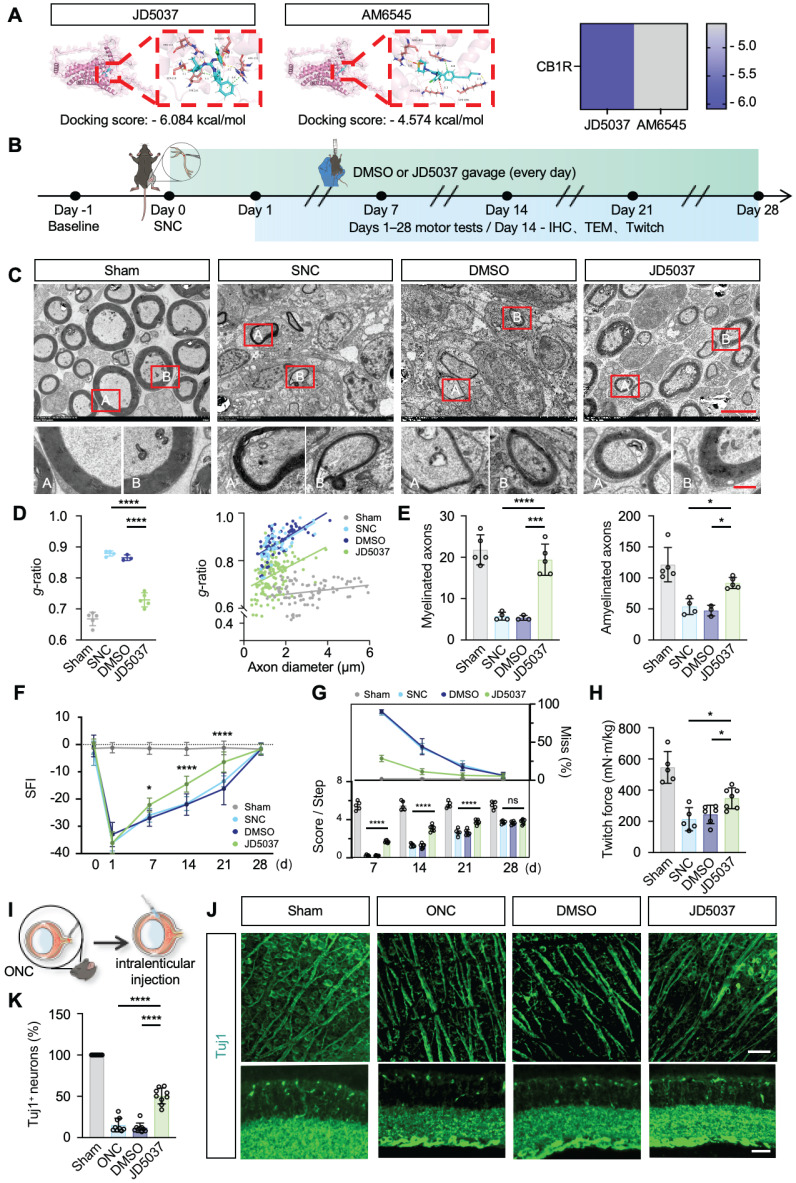
** JD5037 promotes nerve repair by antagonizing CB1R. (A)** Molecular docking analysis of JD5037 and AM6545 with CB1R. JD5037 exhibits a lower binding free energy (ΔΔG = -1.51 kcal/mol). **(B)** Schematic diagram of the experimental workflow. Mice underwent sciatic nerve crush injury and received daily intraperitoneal injections of JD5037 (10 mg/kg) or DMSO for 14 days. Motor function tests were performed from days 1 to 28 post-injury. Tissue collection, IHC, TEM and twitch force measurement were conducted on day 14. **(C, D, E)** TEM images of remyelinated axons in the distal sciatic nerve. JD5037-treated mice displayed more intact myelin sheaths, increased myelin thickness, and a significantly higher number of myelinated axons compared to the SNC and DMSO groups. Red boxes indicate magnified regions. Scale bars = 5 μm (upper), 1 μm (lower). n = 5 in Sham and JD5037 groups. n = 4 in SNC group. n = 3 in DMSO group. **(F)** Quantification of SFI scores of mice in each group. n = 6 in Sham group. n = 7 in SNC and DMSO groups. n = 8 in JD5037 group. **(G)** Quantification of score per step and the percentage of step miss in ladder rung walking test. n = 5 in Sham and SNC group. n = 6 in DMSO group. n = 7 in JD5037 group. **(H)** Quantification of twitch force after single electrical stimulation. n = 5 in Sham and SNC group. n = 6 in DMSO group. n = 7 in JD5037 group. **(I)** Schematic of ONC model followed by intravitreal injection. **(J)** Representative images of retinal flat mounts (top) and cross-sections (bottom) stained for Tuj1 to label retinal ganglion cells (RGCs) and axons in each group: Sham, ONC, DMSO vehicle, and JD5037 treatment. **(K)** Quantification of Tuj1-positive areas in the retina across groups (n = 9). All data are presented as mean ± SD. Statistical analysis: two-way ANOVA with Bonferroni's post hoc test (F, G), one-way ANOVA with Tukey's post hoc test (D, E, H, K). ^*^p < 0.05, ^***^p < 0.001, ^****^p < 0.0001. CB1R, cannabinoid receptor 1; SNC, sciatic nerve crush; ONC, optic nerve crush; DMSO, dimethyl sulfoxide; TEM, transmission electron microscopy; IHC, immunohistochemistry; SFI, sciatic functional index; IF, immunofluorescence.

CB1R has emerged as a promising therapeutic target, with preliminary clinical studies exploring peripheral-selective antagonists such as JD5037 and AM6545 46. JD5037, a reversible peripheral CB1R antagonist currently in early clinical trials, contrasts with AM6545, an irreversible antagonist theoretically offering prolonged efficacy 47,48. Our molecular docking analyses revealed significantly lower binding free energy between JD5037 and CB1R compared to AM6545 (ΔΔG = -1.51 kcal/mol; Figure 7A), indicating higher affinity and potentially superior therapeutic efficacy of JD5037. Thus, we selected JD5037 as our candidate therapeutic compound and evaluated its efficacy in a mouse model of sciatic nerve injury (Figure 7B). Administration of JD5037 (10 mg/kg/day) for 14 days post-injury significantly enhanced axonal regeneration distance, remyelination levels, and overall axonal regeneration range compared to the SNC control group (Figure S3B-C and Figure 7C-E). Immunofluorescent staining further demonstrated that JD5037 treatment significantly improved neuromuscular junction regeneration and endplate morphology (Figure S3D-E). Functional assessments showed marked improvements in motor function in JD5037-treated mice, with significant enhancements observed in the SFI, footprint analysis, and horizontal ladder tests compared to the SNC group (Figure 7F-G). Additionally, muscle contraction force measurements following single electrical stimulation confirmed the beneficial effects of JD5037 on muscle functional recovery (Figure 7H). Preliminary, mechanistic exploration revealed that LC3BII/I, PINK1, and Parkin were upregulated, whereas TOM20 and p62 were downregulated, as shown by Western blot analysis. These findings suggest that JD5037 may promote nerve repair by activating the PINK1-Parkin-mediated mitophagy pathway (Figure S3F-G).

Previous studies have shown that CB1R is expressed in the optic nerve and implicated in ocular diseases 49-52. To assess JD5037's effect on central nerve injury, we used an optic nerve crush model and delivered JD5037 via intravitreal injection (local injection to reach optical nerve) (Figure 7I). Immunofluorescence staining using Tuj1 to label nerve fibers and retinal ganglion cells revealed that, one week after treatment, the number of retinal ganglion cells significantly increased and nerve fibers appeared denser (Figure 7J-K). This dual action expands the application of CB1R antagonists and offers a promising strategy for precise nerve injury treatment.

## Discussion

**Figure 8:**
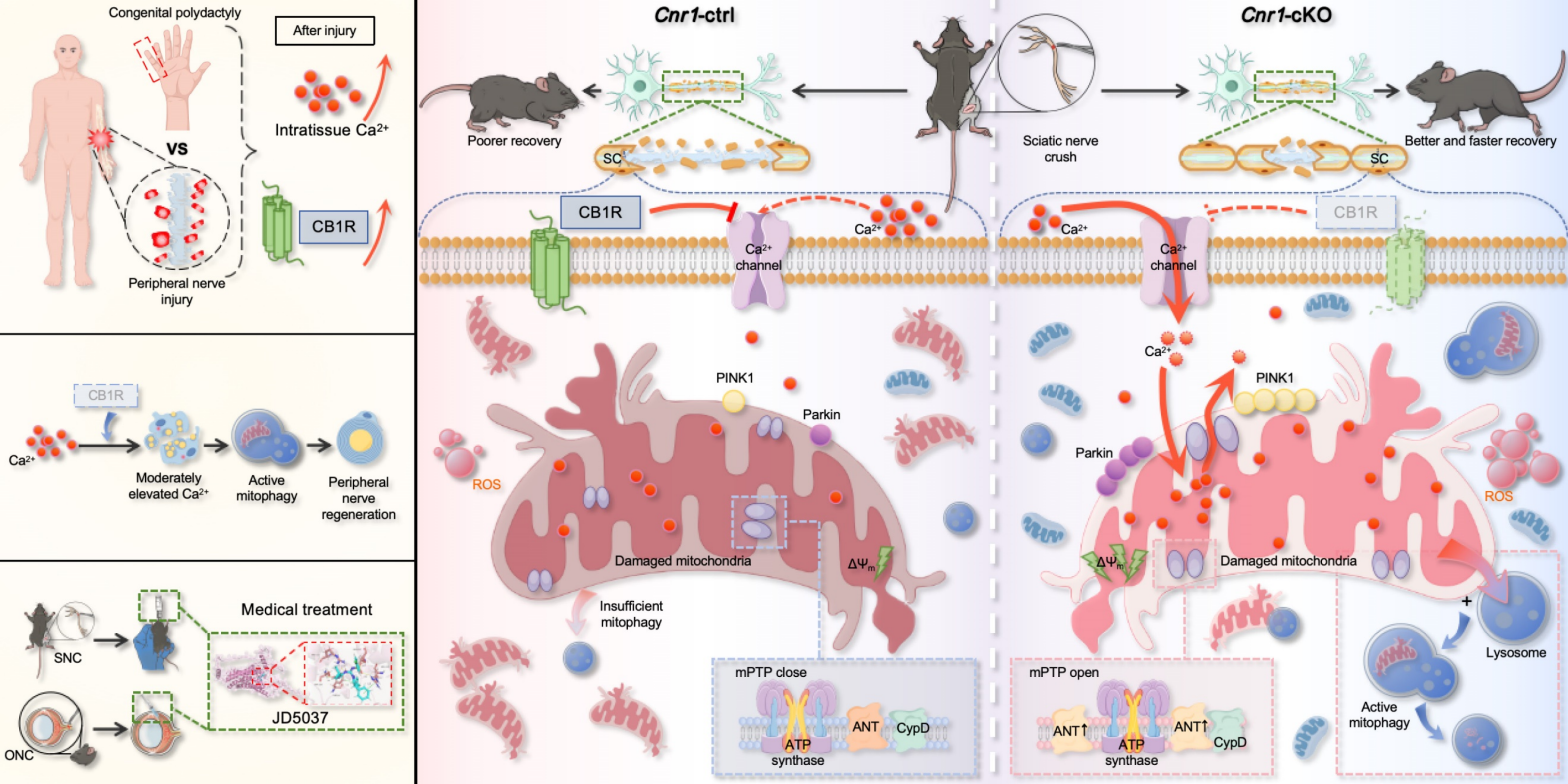
** Schematic diagram illustrating CB1R-mediated regulation of calcium-induced mitophagy and its role in promoting nerve regeneration.** Following peripheral nerve injury, nerve tissue analysis revealed a moderate increase in extracellular calcium concentration within the injured microenvironment, accompanied by upregulation of CB1R expression (top left). Overall, CB1R-mediated regulation of calcium-dependent mitophagy plays a key role in promoting peripheral nerve regeneration (middle left). In addition, the CB1R antagonist JD5037 mimics the effects of genetic deletion and exhibits significant neuroregenerative potential in both sciatic nerve crush (SNC) and optic nerve crush (ONC) models, suggesting promising translational value (bottom left). Mechanistically (right): In control mice (*Cnr1-*ctrl), CB1R is upregulated in Schwann cells after injury, suppressing calcium channel opening and limiting extracellular Ca^2+^ influx. This inhibition impairs Ca^2+^-dependent mitophagy, leading to the accumulation of damaged mitochondria, reduced Schwann cell repair capacity, and delayed axonal regeneration. In contrast, in CB1R-deficient mice (*Cnr1*-cKO), the absence of CB1R removes the inhibitory effect on calcium channels, allowing extracellular Ca^2+^ to enter Schwann cells. Concurrently, CB1R deletion enhances ANT2 expression, which activates mitophagy by PINK1/Parkin pathway, thereby efficiently clearing dysfunctional mitochondria in Schwann cells and accelerating axonal regeneration.

Peripheral nerve regeneration relies on extensive tissue remodeling distal to the injury site, primarily driven by Schwann cells (SCs) 10,53. Upon injury, SCs rapidly transition into a repair phenotype, migrate toward the lesion, and form Büngner bands that support axonal regrowth. This study found that a regenerative microenvironment characterized by a moderate elevation in extracellular Ca2+ levels was formed after nerve injury both in humans and mice. By employing a mouse model, we further confirmed that this environment enhances the repair functions of SCs. Notably, we identify CB1R as a key negative regulator of this process. Injury-induced CB1R upregulation suppresses Ca^2+^ influx, disrupting calcium homeostasis and impairing regeneration. Mechanistically, CB1R deficiency promotes Ca^2+^ influx, upregulates ANT2 expression, and, by modulating the sensitivity of mPTP opening, leads to a decrease in mitochondrial membrane potential. This, in turn, activates the PINK1/Parkin pathway, enhances mitophagy, and ultimately promotes axonal regeneration and functional recovery. These findings demonstrate that CB1R knockout induces mitophagy via the Ca^2+^-PINK1/Parkin signaling axis, thereby promoting peripheral nerve regeneration. Meanwhile, the CB1R antagonist JD5037 exhibited beneficial effects in both peripheral and optic nerve injuries, further highlighting its potential as a promising therapeutic candidate (Figure 8).

Previous studies have shown that calcium ion levels significantly increase following peripheral nerve injury, correlating with the severity of nerve damage 12. Using a rat sciatic nerve compression model, Yan et al. observed a progressive rise in calcium levels within injured nerve fibers at 2, 4, 8, 12, and 24 weeks post-injury, peaking between weeks 2 and 8 13. However, this study did not examine early calcium dynamics, even though Wallerian degeneration initiates immediately after PNI, leaving a critical gap in our understanding of early-stage calcium signaling. Studies using Mito-GCaMP2 and Cyto-GCaMP2 adeno-associated viruses (AAVs) to label myelinating Schwann cells have demonstrated, through *in vivo* real-time fluorescence imaging, that cytosolic calcium oscillates within 1 h after nerve crush and returns to baseline within 2 h 54. In contrast, mitochondrial calcium initially decreases at 1 h but continues to increase beyond the 2-h mark, suggesting a transient cytosolic calcium fluctuation followed by sustained mitochondrial calcium accumulation. However, this study only tracked calcium dynamics within 5 h post-injury. Here, we confirm that calcium levels in the microenvironment increase as early as 1-day post-injury and persist for at least 14 days. Early studies have reported that increased calcium levels reduce the number of SCs. In contrast, moderate calcium upregulation supports Schwann cell survival and function, as demonstrated in primary SCs and sciatic nerve explants in this study.

In clinical practice, previous studies have explored the use of external interventions to modulate calcium channel activity and promote nerve repair, with electrical stimulation (ES) being one of the most representative approaches 14,55,56. ES induces a substantial influx of sodium and calcium ions into neurons, thereby generating action potentials that retrogradely propagate along the axon to the soma—a process that physiologically mimics the natural response following nerve injury 11,14,15. The influx of calcium plays a pivotal role in nerve regeneration, as studies have shown that blocking calcium entry markedly impairs the regenerative response of injured neurons 57. Moreover, externally applied electric fields (1 Hz, 5 V/cm) can promote calcium influx in Schwann cells, thereby upregulating the expression of neurotrophic factors such as nerve growth factor (NGF) and neurotrophin-3 (NT-3) 16. In cultured spinal neurons, ES (20 Hz, 3-5 V, 100 μs pulse width) significantly enhances the mRNA expression of brain-derived neurotrophic factor (BDNF) and its high-affinity receptor, tyrosine kinase B (TrkB), in response to increased intracellular calcium levels 58. Collectively, these findings suggest that ES enhances the repair capacity of neurons and Schwann cells by modulating calcium signaling pathways. Similar to the CB1R-targeted strategy proposed in this study, both approaches converge on the regulation of calcium influx, indicating potential mechanistic synergy and broad clinical applicability for CB1R-targeted therapy in nerve regeneration.

Calcium ions, as a key second messenger, play a crucial role in cellular signal transduction, enzymatic activity regulation, and various physiological processes 59. However, directly modulating intracellular Ca^2+^ levels can trigger aberrant cellular responses, disrupt intracellular homeostasis, and even lead to cell damage or programmed cell death. Consequently, previous studies have primarily adopted indirect approaches to regulate calcium signaling, such as targeting calcium channels with specific drugs 13,60, modulating neuronal Ca^2+^ levels 61, or using novel materials to deliver calcium-responsive molecules 62. Similarly, in this study, we identified CB1R as an upstream regulatory factor capable of precisely modulating calcium signaling pathways. CB1R regulates intracellular Ca^2+^ homeostasis in a cell-specific manner across different cell types, providing a more precise and efficient approach to harnessing calcium signaling following peripheral nerve injury and offering a novel strategy for enhancing nerve repair.

CB1R has been extensively studied in the central nervous system, where it predominantly localizes at presynaptic terminals and modulates the release of neurotransmitters such as GABA, glutamate, and dopamine. Through this mechanism, CB1R plays a critical role in maintaining the excitatory-inhibitory balance, regulating synaptic plasticity, and influencing learning and memory processes 63-65. Additionally, CB1R exerts neuroprotective effects by attenuating glutamate-induced excitotoxicity and oxidative stress 66. Existing research on CB1R in the peripheral nervous system (PNS) primarily focuses on its role in neuropathic pain modulation and metabolic regulation 22,67. Studies have shown that selective activation of peripheral CB1R effectively alleviates neuropathic pain without causing CNS side effects. In a rat sciatic nerve injury model, CB1R agonists exhibit significant analgesic effects without inducing motor impairment or hypothermia 68. In terms of metabolic regulation, peripheral CB1R antagonists have been shown to effectively improve obesity and related metabolic disorders 69. Moreover, our previous study found that abnormal CB1R activation in the PNS is closely linked to excessive ketone production in diabetic neuropathy, and blocking CB1R can mitigate this pathological condition 22. This study uncovers a novel peripheral mechanism of CB1R, demonstrating that it suppresses Schwann cell mitophagy via intracellular Ca^2+^ signaling, thereby hindering peripheral nerve regeneration after injury. This discovery expands our understanding of CB1R function and provides new insights into potential therapeutic strategies for peripheral nerve injuries. In this study, we did not specifically assess the function of mitochondrial membrane-localized CB1R. However, previous research has shown that activation of mitochondria-localized CB1R (mtCB1R) inhibits respiratory chain complex I activity, reducing oxygen consumption and ATP production, thereby negatively regulating cellular energy metabolism 70. This mechanism supports our notion that CB1R inhibition facilitates the maintenance of mitochondrial quality.

In our previous study, our co-authors demonstrated that Parkin-mediated mitophagy effectively reduces mitochondrial ROS production, stabilizes mitochondrial membrane potential, maintains autophagic flux, and inhibits mitochondrial apoptosis, thereby promoting peripheral nerve repair 43. In this study, we further revealed that CB1R inhibition induces mitophagy via the Ca^2+^-ANT2-PINK1/Parkin axis, facilitating peripheral nerve regeneration. This finding aligns with the recently proposed concept that ANT promotes mitophagy during bioenergetic collapse by stabilizing PINK1 through the inhibition of the presequence translocase TIM23 71. Although mitophagy has been widely studied, existing research has primarily focused on its role in neurodegenerative diseases such as Parkinson's disease 72. Upon mitochondrial damage, PINK1 phosphorylates and activates the E3 ubiquitin ligase Parkin, leading to the ubiquitination and clearance of impaired mitochondria, thereby preserving mitochondrial function and cellular homeostasis 73. Additionally, the ANT2-PINK1/Parkin pathway regulates TBK1 subcellular localization, preventing aberrant cell cycle progression and neuronal injury; its disruption contributes to mitochondrial dysfunction and neurodegeneration 74. This study further elucidates the mechanism of mitophagy in SCs and reveals the critical link between mitochondrial dynamics and nerve regeneration. Although proteomic analysis and Western blot results indicate that CB1R mediates calcium-dependent regulation of ANT2 expression, the precise regulatory mechanism remains unclear. Therefore, further research is needed to explore their potential interactions in Ca^2+^ transmembrane transport and mitophagy, providing deeper insights into their functional interplay. Of course, we do not discount the role of intracellular calcium stores (the endoplasmic reticulum) in maintaining calcium homeostasis, but this study primarily focuses on the processes occurring within mitochondria.

Moreover, our findings validate the therapeutic potential of JD5037, a peripheral-selective CB1R antagonist, as a promising strategy for treating nerve injury 47. By restricting blood-brain barrier permeability, JD5037 effectively minimizes the side effects on the central nervous system, underscoring its clinical feasibility. However, we acknowledge several potential limitations and challenges, particularly concerning translation into human applications. The substantial heterogeneity between animal injury models and human neuropathic conditions, such as diabetic neuropathy or traumatic nerve injuries, necessitates cautious interpretation and further validation of these findings. Additionally, whether JD5037 exerts its therapeutic effects through Ca^2+^ signaling *in vivo* remains an open question that warrants further investigation. Finally, comprehensive preclinical and clinical studies assessing JD5037's safety, efficacy, dosage dependency, long-term outcomes, and potential adverse effects will be essential to facilitate its successful clinical translation.

In summary, this study proposes that there is a “regenerative window” of calcium signaling between toxicity and therapy, in which a moderate increase in Ca^2+^ levels (~1.5-fold) after peripheral nerve injury promotes Schwann cell survival and axonal regeneration, challenging the traditional notion of calcium toxicity. Furthermore, it identifies CB1R as a negative regulator of calcium signaling that inhibits nerve regeneration by modulating the Ca^2+^-mitophagy pathway, highlighting the crucial role of CB1R in mitochondrial quality control. Finally, it confirms the regenerative potential of the CB1R antagonist JD5037 in promoting both peripheral and central nerve regeneration, offering a novel therapeutic strategy for precise intervention in nerve injury.

## Supplementary Material

Supplementary figures and tables.

## Abbreviations

CB1R: cannabinoid receptor 1
SCs: Schwann cells
PNI: peripheral nerve injury
PNS: peripheral nervous system
Ca^2+^: calcium
GEO: Gene Expression Omnibus
ANT2: adenine nucleotide translocase 2
PINK1: PTEN-induced kinase 1
Parkin: E3 ubiquitin-protein ligase Parkin
SFI: sciatic functional index
TEM: transmission electron microscopy
IF: immunofluorescence
IHC: immunohistochemistry
CTB: cholera toxin subunit B
DRG: dorsal root ganglion
TA: tibialis anterior
GA: gastrocnemius
NMJs: neuromuscular junctions
α-BTX: alpha-bungarotoxin
NFL: neurofilament
Syn: synaptophysin
mPTP: mitochondrial permeability transition pore
ROS: reactive oxygen species
JC-1: 5,5′,6,6′-tetrachloro-1,1′,3,3′-tetraethylbenzimidazolylcarbocyanine iodide
TOM20: translocase of outer mitochondrial membrane 20
LC3B: microtubule-associated protein 1 light chain 3 beta
DMSO: dimethyl sulfoxide
SNC: sciatic nerve crush
ONC: optic nerve crush
TAM: tamoxifen
GSEA: Gene Set Enrichment Analysis
GO: Gene Ontology
KEGG: Kyoto Encyclopedia of Genes and Genomes
STRING: Search Tool for the Retrieval of Interacting Genes/Proteins
