# Supplementary material for: Targeting CB1R Rewires Ca2+-Dependent Mitophagy to Promote Nerve Regeneration
Source: Theranostics. 2025 Aug 11;15(17):8873–96. doi: 10.7150/thno.119712 (PMC12439258; doi:10.7150/thno.119712)
Supplement: Supplementary file 1 — Supplementary figures and tables. [file thnov15p8873s1.pdf]

Supplementary Figures

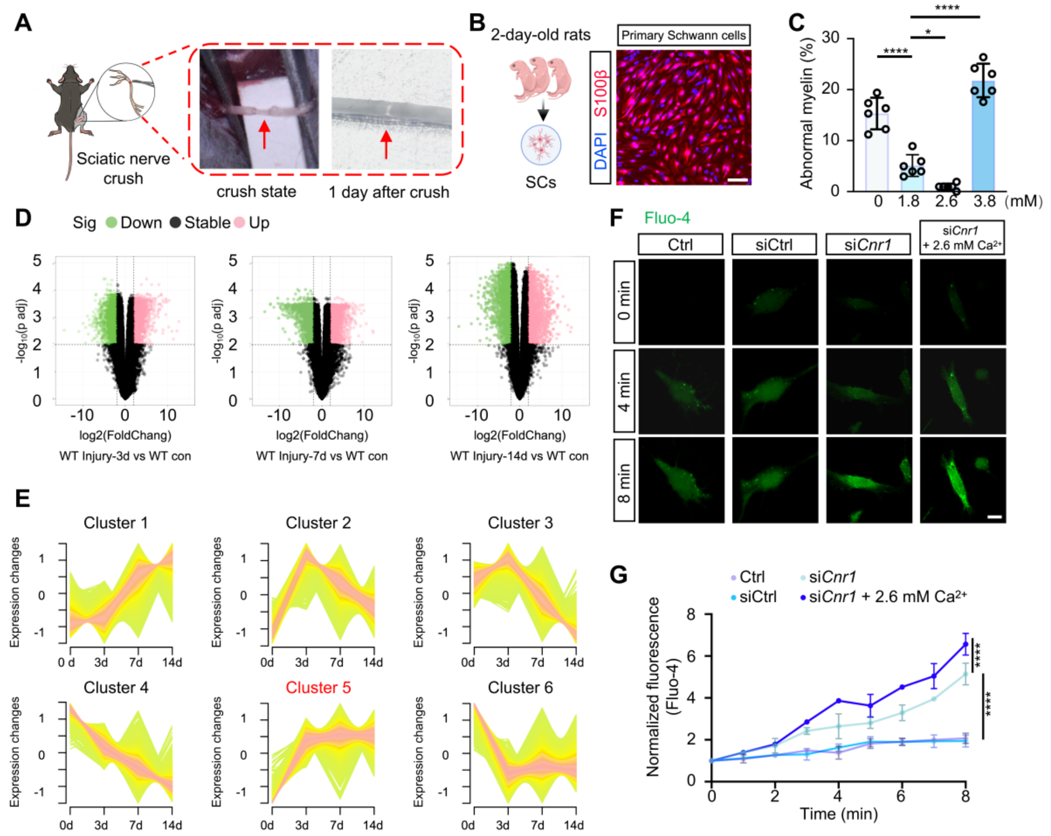

Figure S1. Establishment of the peripheral nerve injury model, Schwann cell isolation, and transcriptomic analysis. (A) Schematic diagram illustrating the sciatic nerve crush injury model. The left panel shows the procedure of crush injury, and the right panel shows the injured sciatic nerve at 1 day post-injury. (B) Workflow of primary SC isolation from sciatic nerves of 2-day-old rats. Representative IF image shows SCs stained with S100β (red) and DAPI (blue), confirming high purity. Scale bar = 10 μm. (C) Quantification of abnormal myelination in sciatic nerve explants treated with varying extracellular Ca^{2+} concentrations (0 mM, 1.8 mM, 2.6 mM, 3.8 mM). n = 6. (D) Volcano plots showing differentially expressed genes (DEGs) at 3, 7, and 14 days post-injury relative to uninjured control (WT con). Upregulated genes are shown in pink, downregulated genes in green, and non-significant genes in black. (E) Mfuzz clustering of gene expression patterns at 0, 3, 7, and 14 days post-injury. Cluster 5 (highlighted in red) displays strong association with injury-induced calcium changes. (F) Fluo-4 fluorescence imaging of intracellular Ca^{2+} levels in CB1R-knockdown Schwann cells (si*Cnr1*), under conditions including Ctrl, siCtrl, si*Cnr1*, and si*Cnr1* + 2.6 mM Ca^{2+}, at 0, 4, and 8 min. Scale bar = 10 μm. (G) Quantification of Fluo-4 fluorescence intensity indicating intracellular Ca^{2+} dynamics across experimental groups. n = 3. All data are presented as mean ± SD. Statistical analysis was performed using one-way ANOVA followed by Tukey' s post hoc test (C), and two-way ANOVA followed by Bonferroni' s post hoc test (G). $^{*}p < 0.05$, $^{***}p < 0.001$, $^{****}p < 0.0001$.

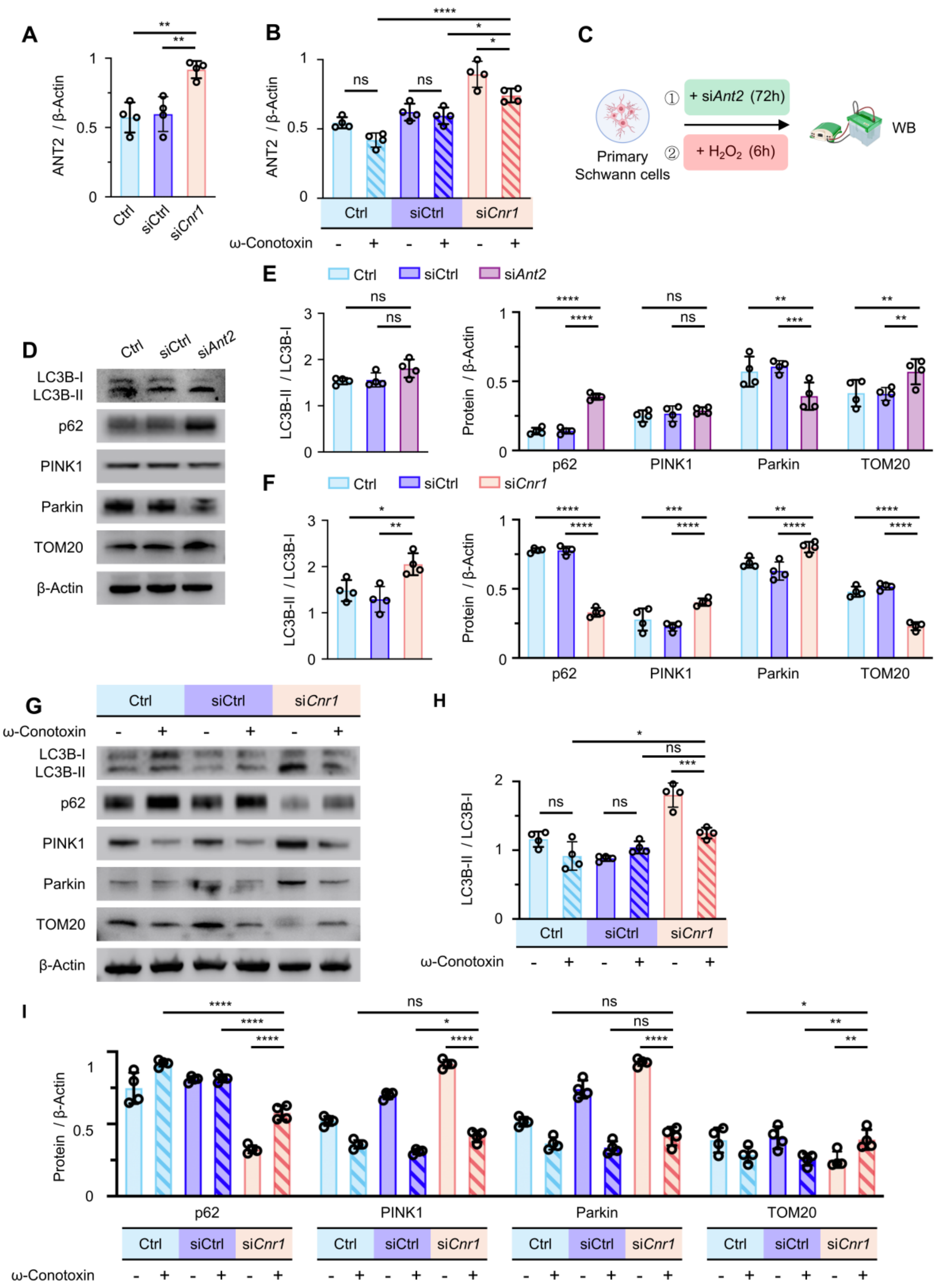

Figure S2. CB1R regulates ANT2 expression and PINK1/Parkin-mediated mitophagy in Schwann cells. (A, B) Quantification of relative expression of ANT2 in Figure 4H and 4I. n = 4. (C) Primary Schwann cells were treated with si*Ant2* for 72 h, followed by H_2O_2 for 6 h, and then subjected to western blot analysis. (D) Western blot analysis of mitophagy-related proteins (LC3B, p62, PINK1, Parkin, and TOM20) after silencing of *Ant2*. (E) Quantification of relative expression of LC3BII/I, p62, PINK1 and Parkin after silencing of *Ant2*. n = 4. (F) Quantification of relative expression of LC3BII/I, p62, PINK1 and Parkin after silencing of *Cnr1* as shown in Figure 5I. n = 4. (G) Western blot analysis of mitophagy-related proteins after treatment with ω-Conotoxin or saline in indicated groups. (H, I) Quantification of relative expression of LC3BII/I, p62, PINK1, Parkin, and TOM20 in (G). n = 4. All data are presented as mean ± SD. Statistical analysis was performed using one-way ANOVA followed by Tukey' s post hoc test. $^{*}p < 0.05$, $^{**}p < 0.01$, $^{***}p < 0.001$, $^{****}p < 0.0001$; ns, not significant.

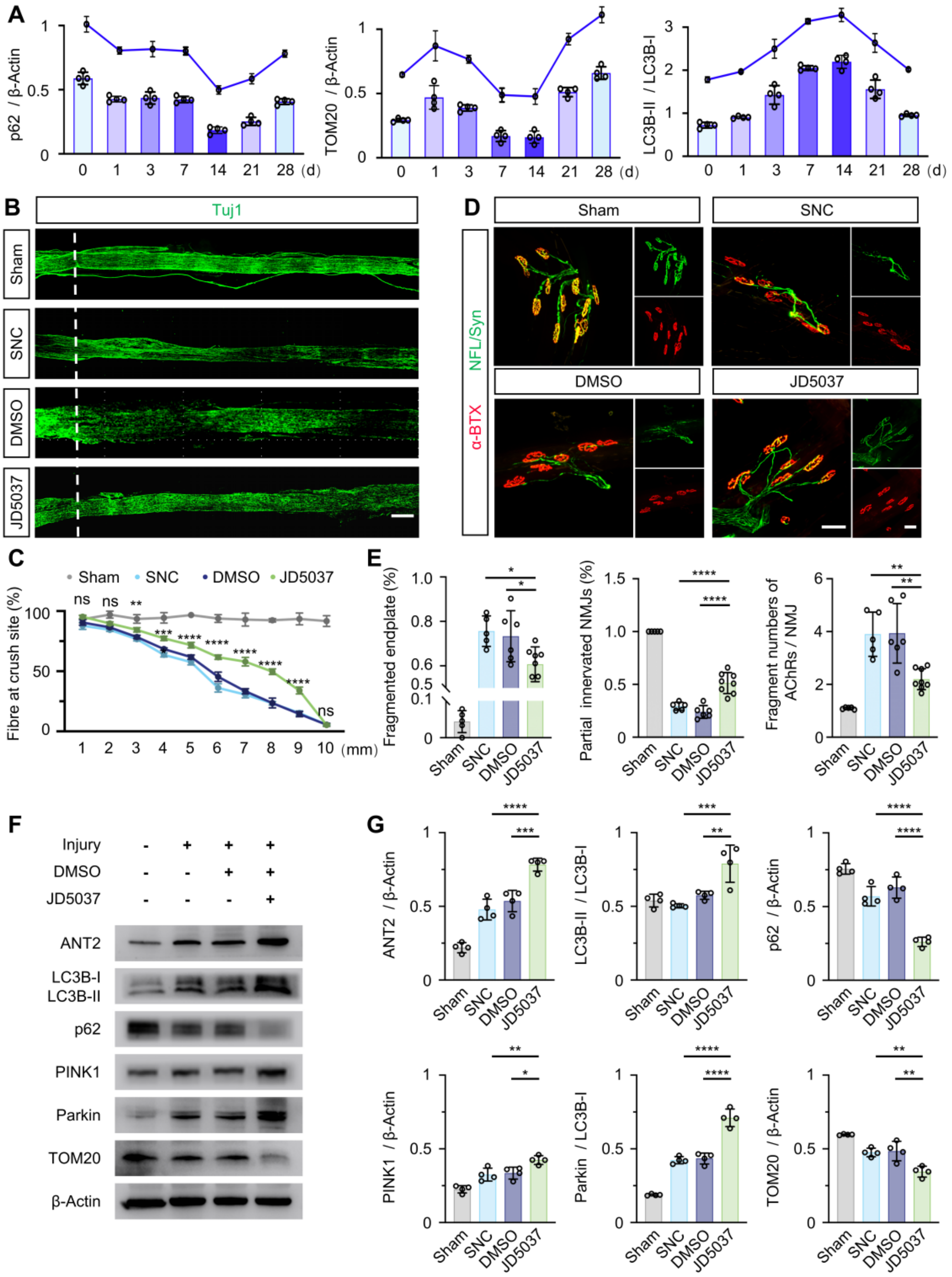

Figure S3. JD5037 promotes axonal regeneration, neuromuscular junction repair, and PINK1/Parkin-mediated mitophagy. (A) Quantitative analysis of relative expression of p62, TOM20 and LC3BII/I in the distal sciatic nerve stump at 0, 1, 3, 7, 14, 21, and 28 days post-injury, as shown in Figure 6B. (B) IF staining of regenerating axons in the distal sciatic nerve labeled with Tuj1 (green). Scale bar = 1 mm. (C) Quantification of axon regeneration at 3–9 mm distal to the injury site at indicated groups. n = 4. (D) IF staining of NMJs with NFL/Syn (green) marking nerve terminals and α-BTX (red) labeling postsynaptic acetylcholine receptors (AChRs). Scale bar = 20 μm. (E) Quantification of fragmented endplates (left), partially innervated NMJs (middle), and fragmented AChRs per NMJ (right) in each group. n = 4. (F) Western blot analyzed the expression of ANT2 and mitophagy-related proteins (LC3B, p62, PINK1, Parkin, TOM20) in sciatic nerve tissues isolated from mice with or without JD5037 administration. (G) Quantification of relative expression of ANT2, LC3BII/I, p62, PINK1, Parkin and TOM20 in (F). n = 4. All data are presented as mean ± SD. Statistical analysis was performed using two-way ANOVA followed by Bonferroni' s post hoc test (C), and one-way ANOVA followed by Tukey' s post hoc test (A, E, G). $^{*}p < 0.05$, $^{**}p < 0.01$, $^{***}p < 0.001$, $^{****}p < 0.0001$; ns, not significant.

Supplementary Tables

Table S1

($[Ca^{2+}]$: Calcium quantification; WB: Western blot)

Category	Patient ID	Sex (M/F)	Age (years)	Sampling Site	Time Since Injury	Assay
Congenital Polydactyly (Con)	P1	M	16	Right digital nerve	–	$[Ca^{2+}]$ & WB
	P2	M	21	Right digital nerve	–	$[Ca^{2+}]$ & WB
	P3	M	18	Left digital nerve	–	$[Ca^{2+}]$ & WB
Severe Peripheral Nerve Injury (PNI)	P4	M	31	Right digital nerve	5.5 h	$[Ca^{2+}]$ & WB
	P5	M	22	Right ulnar nerve	6 h	$[Ca^{2+}]$ & WB
	P6	M	15	Right digital nerve	3 h	$[Ca^{2+}]$ & WB

Table S2

Antibodies	Source	Identifier
Mouse Anti-S100β	Sigma	Cat#S2532
Mouse Anti-Tuj1	Abcam	Cat#ab78078
Rabbit Anti-Synapsin-1	Cell Signaling Technology	Cat#5297
Rabbit Anti-NFL	Cell Signaling Technology	Cat#2837
Mouse Anti-β-Actin	Transgen	Cat#HC201
Rabbit Anti-Cannabinoid Receptor 1	Abcam	Cat#ab3558
Rabbit Anti-NeuN	Cell Signaling Technology	Cat#24307
Rabbit Anti-ANT2	Proteintech	Cat#83104-1-RR
Monoclonal Anti-Thy1.1	Sigma	Cat#M7898
Rabbit Anti-PINK1	Proteintech	Cat#23274-1-AP
Rabbit Anti-PARK2/Parkin	Proteintech	Cat#14060-1-AP
Rabbit Anti-TOM20	Proteintech	Cat#11802-1-AP
Mouse Anti-LC3B	Cell Signaling Technology	Cat#83506
Rabbit Anti-p62(SQSTM1)	MBL International	Cat#PM045
Alexa Fluor 488 Donkey Anti-Mouse	Invitrogen	Cat#A32766
Alexa Fluor 488 Donkey Anti-Rabbit	Invitrogen	Cat#A11008
Alexa Fluor 546 Donkey Anti-Mouse	Invitrogen	Cat#A10040
Alexa Fluor 546 Donkey Anti-Rabbit	Invitrogen	Cat#A11035
Alexa Fluor 647 Goat Anti-Mouse	Invitrogen	Cat#A31571
HRP-Donkey-anti-rabbit	Beyotime	Cat#A0208
HRP-Donkey-anti-mouse	Beyotime	Cat#A0216

Table S3

siRNA sequences		
CNR1	Sense	5'-GGGAAGAUGAACAAGCUUAUC-3
	Anti-sense	5'-UAAGCUUGUUCAUCUUCCCGA-3'
ANT2	Sense	5'-CCUUCAAAGAUAAAUACAAGC-3
	Anti-sense	5'-UUGUAUUUAUCUUUGAAGGCG-3'
Negative	Sense	5'-UUCUCCGAACGUGUCACGUTT-3
	Anti-sense	5'-ACGUGACACGUUCGGAGAATT-3'

Table S4

Chemicals, peptides, recombinant proteins, and Reagent Resource		
JD5037	MedChemExpress	Cat#HY-18697
Tris HCl	Beyotime	Cat#ST772
BAPTA	MedChemExpress	Cat# HY-100168
DMEM/F12	Bioscience	Cat#3130-0500
$CaCl_2$	Sigma	Cat#C5670
Corn Oil	MedChemExpress	Cat#HY-Y1888
Opti-MEM	Gibco	Cat#31985-070
Tamoxifen	MedChemExpress	Cat#HY-13757A
Mdivi-1	MedChemExpress	Cat#HY-15886
Cy3-α-Bungarotoxin	Biotium	Cat#90-1022
ω-Conotoxin GVIA/MVIIC TFA salt	Aladdin	Cat#274837
Rhod-2 AM	Abcam	Cat#142780
DAPI	Genview	Cat#GD3410
Hochest 3342	Solarbio	Cat#23491
Poly-L-lysine Hydrobromide	Sigma	Cat#P8954
Forskolin	Sigma	Cat#F6886
Recombinant Human NRG1-β1	R&D	Cat#377-HB-050
Collagenase from Clostridium histolyticm	Sigma	Cat#C0130
Complement, Rabbit Serum	Calbiochem	Cat#3386909
Fetal Bovine Serum	Sigma	Cat#F8318
1XPBS	Bioss	Cat#C01-01001
1XTBST	Servicebio	Cat#G2150
NGF	Sino Biological	Cat#11050-HNAC
Protease inhibitor mixture	Sangon Biotech	Cat# C50008
Poly-L-ornithine hydrobromide	Sigma	Cat#P3655
Lipofectamine 3000	Invitrogen	Cat#L3000150

Bovine serum Albumin	Genview	Cat#FA016
Donkey serum	Servicebio	Cat#G1217
Goat serum	Servicebio	Cat#G1208
DMSO	Sigma	Cat#D2650
Penicillin-Streptomycin Solution	Beyotime	Cat#C0222
Multicolor Prestained Protein Ladder	EpiZyme	Cat#WJ103
Tween-20	Ding Guo	Cat#DH358-3
Tween-80	Ding Guo	Cat#DH360-3
PEG-300	Shyuanye	Cat#25322-68-3
TritonX-100	Ding Guo	Cat#DH351-4
Western Blot Antibody Stripping Solution	Epizyme	Cat#PS107

Table S5

**Critical commercial assays**		
Calcium Colorimetric Assay Kit	Beyotime	Cat#S1063S
Fluo-4 Calcium Fluorometric Assay Kit	Elab Science	Cat#E-BC-F100
ROS Assay Kit	Beyotime	Cat#S0033S
Mitochondrial Permeability Transition Pore Assay Kit or MPTP Assay Kit	Beyotime	Cat#C2009S
TB Green™ Premix Ex Taq™	TaKaRa	Cat#RR420A
CCK-8 Assay Kit	DoJinDo	Cat#CK04
JC-1	Beyotime	Cat#C2006
Tissue or Cell Total Protein Extraction Kit	Sangon Biotech	Cat#C510003
